# Factors related to the change in Swiss inpatient costs by disease: a 6-factor decomposition

**DOI:** 10.1007/s10198-020-01243-3

**Published:** 2021-01-12

**Authors:** Michael Stucki

**Affiliations:** 1 Winterthur Institute of Health Economics, Zurich University of Applied Sciences, Gertrudstrasse 15, 8401 Winterthur, Switzerland; 2 Department of Health Sciences and Medicine, University of Lucerne, Frohburgstrasse 3, 6002 Lucerne, Switzerland

**Keywords:** Health care costs, Cost-of-illness, Inpatient care, Switzerland, Cost decomposition, I10 Health general, I11 Analysis of health care markets

## Abstract

There is currently little systematic knowledge about the contribution of different factors to the increase in health care spending in high-income countries such as Switzerland. The aim of this paper is to decompose inpatient care costs in the Swiss canton of Zurich by 100 diseases and 42 age/sex groups and to assess the contribution of six factors to the change in aggregate costs between 2013 and 2017. These six factors are population size, age and sex structure, inpatient treated prevalence, utilization in terms of stays per patient, length of stay per case, and costs per treatment day. Using detailed inpatient cost data at the case level, we find that the most important contributor to the change in disease-specific costs was a rise in costs per treatment day. For most conditions, this effect was partly offset by a reduction in the average length of stay. Changes in population size accounted for one third of the total increase, but population structure had only a small positive association with costs. The most expensive cases accounted for the largest part of the increase in costs, but the magnitude of this effect differed across diseases. A better understanding of the factors related to cost changes at the disease level over time is essential for the design of targeted health policies aiming at an affordable health care system.

## Introduction

High and rising health care spending dominates the health policy debate in high income countries. OECD countries have experienced a strong increase in spending in the last few years, raising questions about the sustainability of its levels. Switzerland is one of the countries with the highest share of health care spending in percent of its GDP in the world (12.3% in 2017) [1]. The average annual health care spending growth of 3.7% over the last 25 years outpaced nominal GDP growth (2.3%). Switzerland also exhibited the second largest health care spending per capita after the United States with 9,768 Swiss Francs (about 10,000 US Dollar) in 2017, which is twice the spending in 1992.

The inpatient care sector accounts for about one-fifth of total health care spending in Switzerland [1] and went through some reforms recently. As of 2017, there were 281 hospitals in Switzerland, 102 of which were general hospitals mainly in primary care [2]. In 2013, Switzerland had 4.7 hospital beds per 1000 people, significantly more than the OECD member states average (3.8/1000) [3]. Recent changes include the implementation of a DRG (diagnosis-related groups)-based reimbursement system in 2012, the subsequent structural changes in the service provision (mainly the shift from inpatient to outpatient treatments) as well as the demographic transition which came with changing morbidity patterns. These issues render the inpatient care setting in Switzerland an interesting and relevant case for studying the factors associated with the cost increase at the level of specific diseases.

This study aims to quantify the contributions of six factors related to the change in aggregated inpatient care costs in the Canton of Zurich between 2013 and 2017. The decomposition is done by diseases and age/sex groups. We define a multiplicative relationship between the six factors and apply the rate decomposition method by Das Gupta [4] to separate the impact of (1) population growth, (2) age and sex structure, (3) the number of treated patients, (4) utilization in terms of the number of stays per patient, (5) the length of stay and (6) the costs per day by disease on the disease-specific cost change. The study shows that the increase in costs per day had the strongest effect on the total cost increase of about 15%. Since there was at the same time a reduction in the length of stay that partly offset this effect, our results suggest a compression and increased intensity of inpatient treatments.

Our contribution is threefold. First, in contrast to most related studies (e.g. [5]), we use cost instead of reimbursement data to decompose cost changes over time. In doing so, we focus on the production costs instead of the costs borne by payers. This allows us to distinguish between different components of total costs such as physician costs, medical costs and medical products costs, which enables a more detailed view of the source of cost changes. In addition, it puts more variation into the data compared to if we used the case output price resulting from the case weight calculation. Even though a DRG system aims at applying a model where reimbursement reflects on average production costs, this will not work for all diseases and some of the treatment costs might not be mirrored in the DRG weight. The use of cost data makes sense, as in a dynamic reimbursement system, the DRG weights are, to some extent and in the long run, a function of the production costs. Second, we include an additional factor in the decomposition, namely the length of stay by disease. Thereby, we are able to quantify the contributions of both length of stay and stays per patient. Separating out these factors is important in DRG-based reimbursement schemes, in particular after their introduction. The reason is that decreases in length of stay may be offset by increases in stays per patient if, for example, early discharges come with a higher rate of hospital readmission. Third, we explore cost changes across the full cost distribution. Uncovering differential impacts of the factors across the distribution of case costs by disease may shed light on additional effects that are not visible for the average. To the best of our knowledge, this is the first study that aims to explain the increase in inpatient health care costs with a disease-specific costs approach for Switzerland.

This paper is structured as follows. Section 2 gives background information about the Swiss context and the related literature on health care spending drivers. Section 3 introduces the data and section 4 the methodology. The results are presented in Sect. 5 for the decomposition at two levels of disease classification. Section 6 concludes and discusses the main findings as well as the strengths and limitations of the study.

## Background

### The Swiss Health Care System

Switzerland has universal health coverage with a comprehensive benefits catalogue. It provides high quality health care without waiting times, albeit at high costs [6]. In addition to the basic coverage, the insured can buy supplementary insurance for the inpatient sector, which will provide them higher standard accommodation and treatment by chief physicians. Switzerland is divided into 26 cantons, which partially finance inpatient care services and are responsible for hospital planning. The canton of Zurich with almost 1.5 million inhabitants in 2017 is the biggest canton and home to 25 listed hospitals and six non-listed hospitals. A listed hospital (which may be owned either publicly or privately) receives reimbursement from the cantons (at least 55% of the costs of each inpatient stay that is covered by the mandatory health insurance scheme), whereas non-listed hospitals are exclusively financed by out-of-pocket spending and supplementary insurance. In 2012, Switzerland introduced a new hospital financing and payment system. On the financing side, the dual funding role by the mandatory health insurance and the cantons was standardized across the country. On the payment side, a DRG-based reimbursement scheme was introduced for the acute inpatient sector. This reform put more pressure on the hospitals to provide treatments more efficiently because they now receive case-based lump-sum payments rather than a fee for service reimbursement. Therefore, post-reform hospitals are expected to strive for more efficiency, e.g. by reducing length of stay. At the same time, there is a shift of surgical interventions from the inpatient to the outpatient setting, which tends to reduce inpatient demand and potentially puts financial pressure on hospitals. Total spending for acute inpatient care services increased after the DRG implementation by 4.4% in 2012 and 2013, but the growth slowed down afterwards, with rates between –0.3% (2017) and +2.7% (2015 and 2016) [1].

### Drivers of health care spending

Factors associated with the large increase in health care spending in high-income countries are still not well known. Most of the literature that aims to explain the spending increase looks at one specific factor and does not distinguish between service categories. Examples include the effect of demographic change in age structure and proximity to death (e.g. [7, 8]), the impact of technological progress in medicine (e.g. [9, 10]), incentives faced by providers (supplier-induced demand, e.g. measured by physician density as in [11] for Switzerland), and higher input prices. The latter is sometimes linked to Baumol’s cost disease (empirical evidence for OECD countries including Switzerland see [12] and [13]), stating that input prices (wages for medical staff) increase over-proportionally in labour-intensive sectors such as health care compared to other sectors because productivity gains are only partly possible in these branches.

None of these approaches has taken a disease-based perspective, i.e. variation of these trends across diseases is not taken into account. Some studies have shown the usefulness of disease-based analyses to investigate health care cost drivers. These studies used estimates of disease-specific spending that have been published for some countries (e.g. [14] for the United States, [15] for Norway, [16] for Switzerland). If disease-specific spending estimates are available at different points in time, they can be tracked to identify the changes in the associated factors at a more granular level. Most of the related literature is from and for the United States [17–22]. These studies found for different periods and different populations, that the increase in spending per treated case was the most important factor associated with the spending increase in the last three decades. However, they were mostly limited to two factors, such as the number of (treated prevalent) cases and costs per case.

From a methodological point of view, the two studies that are most closely related to our paper are the two recent decompositions by Zhai et al. for China [23] and Dieleman and colleagues for the United States [5]. Zhai et al. [23] used estimates of health care spending by disease in China for 1993 and 2012 and found that the most important factor associated with the increase was a strong rise in real costs per case. The effect of the change in the age structure was relatively small. Dieleman et al. [5] found similar results for the US. They found that spending per visit (outpatient) and spending per bed-day (inpatient) were the most important factors related to the increase in spending between 1996 and 2013. For inpatient care, they also found a negative association between spending and utilization, which was defined as bed-days per prevalent case, suggesting that the length of stay was reduced. They did, however, not explicitly include this factor. These two studies are able to show the relative importance of five basic factors and to estimate their contributions by disease, using the same methodology and the same disease classification as the present study.

There are only few studies from comparable countries that investigated the drivers of inpatient care costs specifically. Wong et al. [24] assessed the impact of age and proximity to death for disease-specific hospital spending using Dutch data and showed that for more lethal diseases, time to death is much more relevant than age per se. Most of the literature for Switzerland has focused on the (causal) effect of the recent DRG implementation on costs, utilization and quality. It was shown that after the reform in 2012 the return per inpatient case increased only slightly between 2012 and 2015 (though again at a higher pace in 2016) [25]. The most important driver was the increase in the hospitalization rate (on average + 2.1% per year). One study found that age and sex standardized hospitalization rates in acute inpatient care in Switzerland did not change between 2009 and 2016 [26].

## Data

### Overview

The data set was provided by the Department of Health of the canton of Zurich. It holds detailed diagnostic and cost information at the case level for all inpatient acute care episodes in the 25 hospitals listed in the canton of Zurich between 2013 and 2017.^1^ Rehabilitation and psychiatric care facilities are not included in the data. There are between 208,000 and 227,000 inpatient cases per year. A case is defined according to the case consolidation rules in the DRG system; with only few exceptions, any readmission for the same cause within 18 days since the first discharge is recorded in the same administrative case, i.e. a case can consist of multiple single stays. In what follows, the terms case and stay will be used interchangeably. Patients can be tracked over time and across hospitals. This enables the identification of recurrent encounters at the patient level. The data set provides information on patient’s demographics, hospitalization, insurance status, detailed diagnostic information with up to 50 diagnoses per stay according to the International Classification of Diseases, Volume 10 (ICD-10) and detailed case-specific cost data differentiated by 30–40 cost types, depending on the year of observation.

Due to a change in the hospital accounting framework in 2013, we only used data from 2013 to 2017. Not all inpatient cases are reimbursed based on the DRG system. Between 2013 and 2015, about 600 cases per year were reimbursed based on a special palliative care rate. The number of these cases fell to about 100 in 2016 and 2017. We did not exclude non-DRG observations from the data since we were interested in costs rather than reimbursement. For the age/sex group specific population numbers, we used the data from the Statistical Office of the canton of Zurich [28].

### Data manipulations

We assigned each case to one of 42 age/sex groups: 21 age groups at five year age intervals except for the youngest (< 1 year) and the oldest age group (> 95 years) by sex. Since we aimed at tracking the number of unique patients within each disease group for each year, we used the patient identifier based on the anonymized social insurance number that has been available since 2012. This variable showed missing values for all non-resident patients and for some Swiss residents. To fill these gaps, we used a second patient identifier. This identifier uniquely identifies patients within each hospital and year, but not across hospitals and years. Therefore, we slightly overestimate the number of unique patients because some of them might have been treated at different hospitals for the same disease in a given year. Some cases did not fall completely in one calendar year. Patients admitted at the end of a year but without discharge in the same year had no assigned diagnosis and were thus dropped. Some observations with entry and discharge date in the same calendar year were lacking a diagnosis and, thus, had to be excluded from the analysis. About five cases per year with entry in the last year and discharge in the following year (with total length of stay of more than 1 year) were also excluded from the analysis.

### Cost categories

Our data contains rich cost information at the case level. The term *costs* refers to production costs of health services and not to the costs charged to the payer, which is based on the DRG case weight. This is a key innovation of the study, as to our knowledge, there has been no previous literature using the production cost information at the inpatient case level in Switzerland. With the introduction of the DRG system in 2012, hospitals applied a unified accounting standard, ensuring consistent accounting rules across hospitals over the whole period. For each case, costs are recorded and distinguished by about 35 cost types. We aggregated the cost types into cost categories with the support of the Department of Health. Five categories of costs are distinguished: physician costs, medical products costs (e.g. drugs), medical costs (e.g. nursing, diagnostics), other variable costs (e.g. accommodation, transport), and investment or infrastructure costs (depreciation). While the two categories ‘physician costs’ and ‘medical costs’ mainly consist of cost items recorded directly at the case level, the other cost categories comprise fixed costs that are generated through an allocation key. Most hospitals used the same accounting framework (REKOLE^®^ [29]) with strict definitions of the allocation key for each shared service to redistribute costs to cases. The key is closely linked to the actual resource use (e.g. minutes spent with a service) and is not just simply pro rata. REKOLE^®^ defines minimal accounting rules, but hospitals have the possibility to apply a more detailed cost distribution (e.g. by allocating costs according to minutes after reweighting based on case severity or the intensity of service use). We assume that there has been no change in how fixed costs are distributed between 2013 and 2017. The costs comprise both the part covered by the mandatory as well as the supplementary health insurance plan. Details about the mapping of cost types to cost categories as well as the record types are provided in the appendix.

### The Global Burden of Disease Classification

The set of health conditions was defined based on the comprehensive and mutually exclusive Global Burden of Disease (GBD) classification. This classification defines three hierarchical levels of diseases. GBD Level 1 distinguishes between communicable and non-communicable diseases and injuries. Level 2 groups major diseases such as neoplasms or cardiovascular diseases. Level 3 defines more specific diseases such as breast cancer or ischemic heart disease. The ICD-10 codes entailed in our data were mapped to a slightly modified version of the GBD disease classification. We grouped the codes according to the classification used in the 2017 Global Burden of Disease Study to obtain groups of disease codes [30]. To obtain a comprehensive and mutually exclusive classification, we made minor adjustments to the classification.^2^ We generally applied the level 3 classification with some exceptions. All five types of injuries as well as all eight types of communicable diseases were distinguished at level 2 only. The non-communicable diseases were classified at level 3, except for mental disorders and skin and subcutaneous diseases.^3^ We included two more categories for non-diseases/well care and non-distinctive codes. The adapted classification resulted in 100 diseases. The full list of diseases is provided in the appendix.

We allocated all the costs based on the primary diagnosis listed in each record. Accounting for concurrent conditions in cost-of-illness studies is essential and may impact cost estimates significantly [31]. Nevertheless, we did not include any secondary diagnoses in our analysis for three reasons. First, according to the Department of Health, the primary diagnosis coded has to be the diagnosis that caused the most amount of work for the hospital. Second, the impact of comorbidities may differ across different cost categories, i.e. it would be necessary to adjust each of the cost categories for the influence of further diagnoses. Third, there are methodological difficulties associated with such an adjustment, e.g. the unintended incorporation of miscoding or the missing out of further comorbidities at the patient level.

## Methods

### Decomposition of aggregate measures

We used the Das Gupta method for the decomposition of aggregate measures [4] that was further extended for high numbers of factors [32]. The idea of this method is to correct for compositional effects when comparing multiple populations. The observed difference between two groups or two measures from the same group but from different points in time may be due to different characteristics in the two underlying populations. Our method of choice is rather mathematical than econometric such as the Oaxaca–Blinder decomposition approach [33, 34]. The method decomposes the difference between two points in time into its additive components. The contributions of single factors add up to 100% of the total change and no residual remains. The interaction effects arising in the calculation of counterfactuals are distributed among the factors. The decomposition does not depend on the order in which the factors are included in the model. In the standard example, the aggregate measure is a product of the factors.

### 6-Factor decomposition

We decomposed the change in disease-specific inpatient care costs between 2013 and 2017 into the changes caused by the six factors population size, population structure (captured in the model by the distribution of age/sex groups), inpatient treated prevalence (number of unique patients within each age/sex group), utilization (number of stays per prevalent patient), average length of stay (LOS), and costs per day of treatment. Treated prevalence and utilization are actually the two components of the admission rate; the first factor represents the number of patients treated for a certain disease (extensive margin), the second refers to the average number of stays of those who were treated (intensive margin). The costs per day component is a sum over the five cost categories (*c*). We exploited the fact that the aggregate *costs* in one year (2013 or 2017) can be re-written as the sum over the costs observed in each age/sex group (*a*) and disease (*d*) cell. These costs, in turn, are a product of the six factors.

The functional form is given by

1 $$\begin{aligned} costs&= \sum _{d=1}^{100} \sum _{a=1}^{42}population*\frac{population_{a}}{population}\\&\quad* \frac{treated\,patients_{a,d}}{population_{a}} \nonumber *\frac{stays_{a,d}}{treated\,patients_{a,d}}\\&\quad*\frac{total\,LOS_{a,d}}{stays_{a,d}}*(\frac{physician\,costs_{a,d}}{total\,LOS_{a,d}}\\&\quad+\frac{medical\,costs_{a,d}}{total\,LOS_{a,d}}+\frac{med.products\,costs_{a,d}}{total\,LOS_{a,d}}\\&\quad+ \frac{other\,costs_{a,d}}{total\,LOS_{a,d}}+\frac{investment\,costs_{a,d}}{total\,LOS_{a,d}}) \end{aligned}$$

The total cost difference by disease between 2013 and 2017 is the sum of the age/sex group specific rate decompositions. Summing these values up over all diseases gives the total increase in costs between 2013 and 2017.

The 6-factor decomposition does not directly reveal the contribution of the change in costs per case. This key factor is instead captured by costs per day and length of stay. We ran a decomposition including only five factors to explicitly show the impact of the change in costs per case by disease.^4^

### Decomposition by distribution

To allow for a more detailed view on what happens along the full distribution of costs per case, we created five groups of cases for each disease. All cases were allocated to one of these groups based on their location in the distribution of costs per case, and for 2013 and 2017 separately. The five thresholds for the cost groups were: <10th percentile (group 1), between the 10th and the 25th percentile (group 2), between the 25th and the 75th percentile (group 3), between the 75th and the 90th percentile (group 4) and >90th percentile (group 5). The aim of this analysis was to investigate whether the importance of the factors differs across the distribution and which group of cases contributed most to the observed cost change.

For the distributional analysis, we applied a slightly different decomposition specification. As the total effect in the first specification is the sum over all age/sex group and disease-specific cells, the addition of a third dimension (distribution of case costs) would lead to cells with potentially very few observations. In addition, the decomposition would include an additional factor - the share of each age/sex group within each distribution section by disease - which is not easily interpretable. We removed one dimension from the decomposition, namely the population structure. This means that in the second specification, the factors treated prevalence and utilization refer to the whole population (instead of to specific age/sex groups) for each disease. The two factors length of stay and cost per day are distribution group (*g*) specific and are "linked" to the three previous factors by including a technical factor *case share* ($= \frac{{stays_{{g,d}} }}{{stays_{d} }}$), which is the share of the number of cases in each distribution group of the total cases for this disease. By definition, this factor is equal to 10% for the distribution groups 1 (<10th percentile) and 5 (>90th percentile) etc. The specification for the second part of our analysis thus reads as follows:

2 $$\begin{aligned} costs &= \sum _{d=1}^{100} \sum _{g=1}^{5}population*\frac{treated\,patients_{d}}{population}\\&\quad*\frac{stays_{d}}{treated\,patients_{d}}\nonumber *\frac{stays_{g,d}}{stays_{d}}*\frac{total\,LOS_{g,d}}{stays_{g,d}}\\&\quad*(\frac{physician\,costs_{g,d}}{total\,LOS_{g,d}}+\frac{medical\,costs_{g,d}}{total\,LOS_{g,d}}\nonumber \\&\quad+\frac{med.products\,costs_{g,d}}{total\,LOS_{g,d}}+ \frac{other\,costs_{g,d}}{total\,LOS_{g,d}}\\&\quad+\frac{investment\,costs_{g,d}}{total\,LOS_{g,d}}) \end{aligned}$$

The results were obtained by applying the recently implemented STATA package rdecompose [35].

## Results

### Descriptive statistics

Between 2013 and 2017, total acute inpatient costs for all diseases increased from CHF 2.67 billion to CHF 3.06 billion. This corresponds to an increase by CHF 394 million or 14.7%. The most important contributors to this rise were non-communicable diseases. More than 70% of the total cost increase was caused by these diseases, among which cardiovascular diseases had the biggest impact (22.5 percentage points), closely followed by neoplasms (18.2) and musculoskeletal disorders (10.8). 28.3 % of the total cost increase was observed in the treatment of communicable diseases (10.7) and injuries (17.6).

**Fig. 1:**
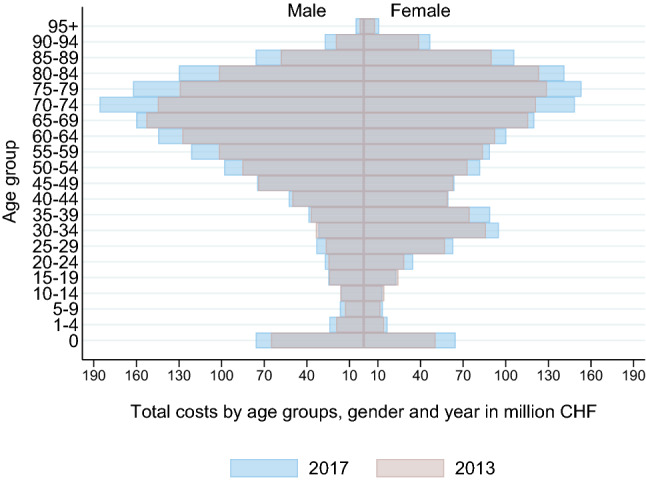
Total costs by age groups, gender and year

**Fig. 2:**
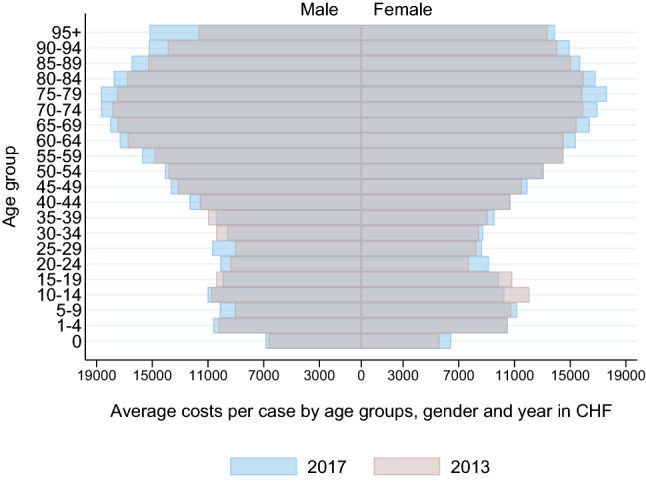
Average costs per case by age groups, gender and year

Figure 1 illustrates the total costs by year stratified by age groups and gender. The highest absolute increase was observed between age 70 and 89 for both men and women. Even though the patients aged 0 represent the smallest age group, the costs and their absolute increase was higher than in most age groups up to 54 years, for both men and women. The average costs per case increase with age, especially between age 40 and 80, as is shown in Fig. 2. The highest absolute increases in costs per case were found for the oldest patients.

Table 1 shows the change in costs per case by cost category as well as the change in average length of stay between 2013 and 2017. In this period, mean costs per case increased by 5.2% to roughly CHF 13,500 in 2017. The largest growth rates were observed for physician costs (+ 7.4%) and other costs (+ 23.5%), but overall these two categories made up less than one-third of the total case costs in 2017. In both years, about half of the total costs per case were medical costs. Here, the growth rate was much lower at 3.4%. The only cost category that showed a decrease between 2013 and 2017 was investment costs per case, but these costs accounted for less than 8% of the total costs per case in both years. The share of fixed costs by disease varied between 70.9% (musculoskeletal disorders) and 92.9% (well care) in 2017. Across all conditions, it was 80.7% in 2017, slightly lower than in 2013 (81.3%). Moreover, there was an increase in the number of cases per year by 9.1%. However, this increase was partly offset by a decrease in the average length of stay. Table 4 in the appendix also shows these statistics by disease group (GBD level 2). Overall, we observe similar patterns across all disease groups, except for few diseases that showed a significant decrease in costs per case (e.g. respiratory infections, substance use disorders, unintentional injuries) or a significantly stronger increase (other infectious diseases). The magnitude of change for the cost categories differs across diseases.

**Table 1:** Summary statistics and growth rates for period 2013–2017

	2013	2017	Change 2013–2017
Total inpatient costs (bn CHF)	2.67	3.06	+ 14.7%
Total Cost per case (CHF)	12804	13466	+ 5.2%
	(22744)	(23427)	
Physician costs per case (CHF)	2755	2959	+ 7.4%
	(4637)	(5161)	
Medical products costs per case (CHF)	1785	1893	+ 6.1%
	(5825)	(5732)	
Medical costs per case (CHF)	6315	6532	+ 3.4%
	(12999)	(13516)	
Other costs per case (CHF)	959	1183	+ 23.5%
	(1243)	(1415)	
Investment costs per case (CHF)	991	899	– 9.1%
	(1609)	(1346)	
Length of stay (LOS) (d)	5.9	5.5	– 6.8%
	(8.0)	(7.3)	
Cases	208438	227436	+9.1%

Mean coefficients; sd in parentheses

### 6-factor decomposition results

**Fig. 3:**
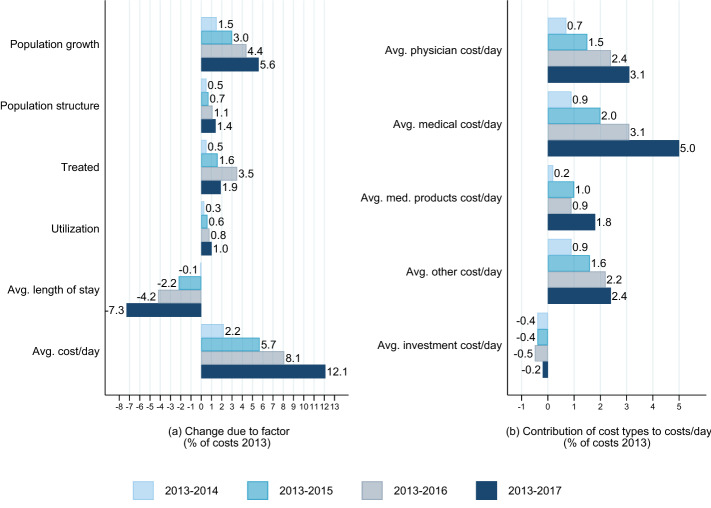
Aggregated 6-factor decomposition results (2013–2017) and detailed decomposition for costs/day factor

Plot a on the left-hand side of Fig. 3 shows the cost change in percent of 2013 costs associated with each of the six factors after decomposing the increase based on 100 diseases and 42 age/sex groups. To assess the consistency of the factors’ relevance across the full period, we also show the associations resulting from similar decompositions for the periods 2013–2014, 2013–2015, and 2013–2016. The increase in population size accounted for about one third of the total nominal increase. The changing population structure only led to a modest increase of total costs, as did the higher proportion of the population that was treated and the utilization factor, i.e. the number of stays per prevalent case. The reduction of the average length of stay was associated with a strong decline in costs. Costs would have decreased by 7.3% if only the length of stay had fallen to the level in 2017 and all the other factors had been as in 2013. The contribution of the costs per day factor is clearly positive (12.1%). Plot b on the right-hand side of Fig. 3 shows the contributions of the five cost types to the 12.1% increase. The biggest contribution (5 percentage points of the total increase) can be observed for the medical cost category. This is not very surprising given that about half of the total costs per case are medical costs which have modestly increased between 2013 and 2017 (see Table 1).

**Fig. 4:**
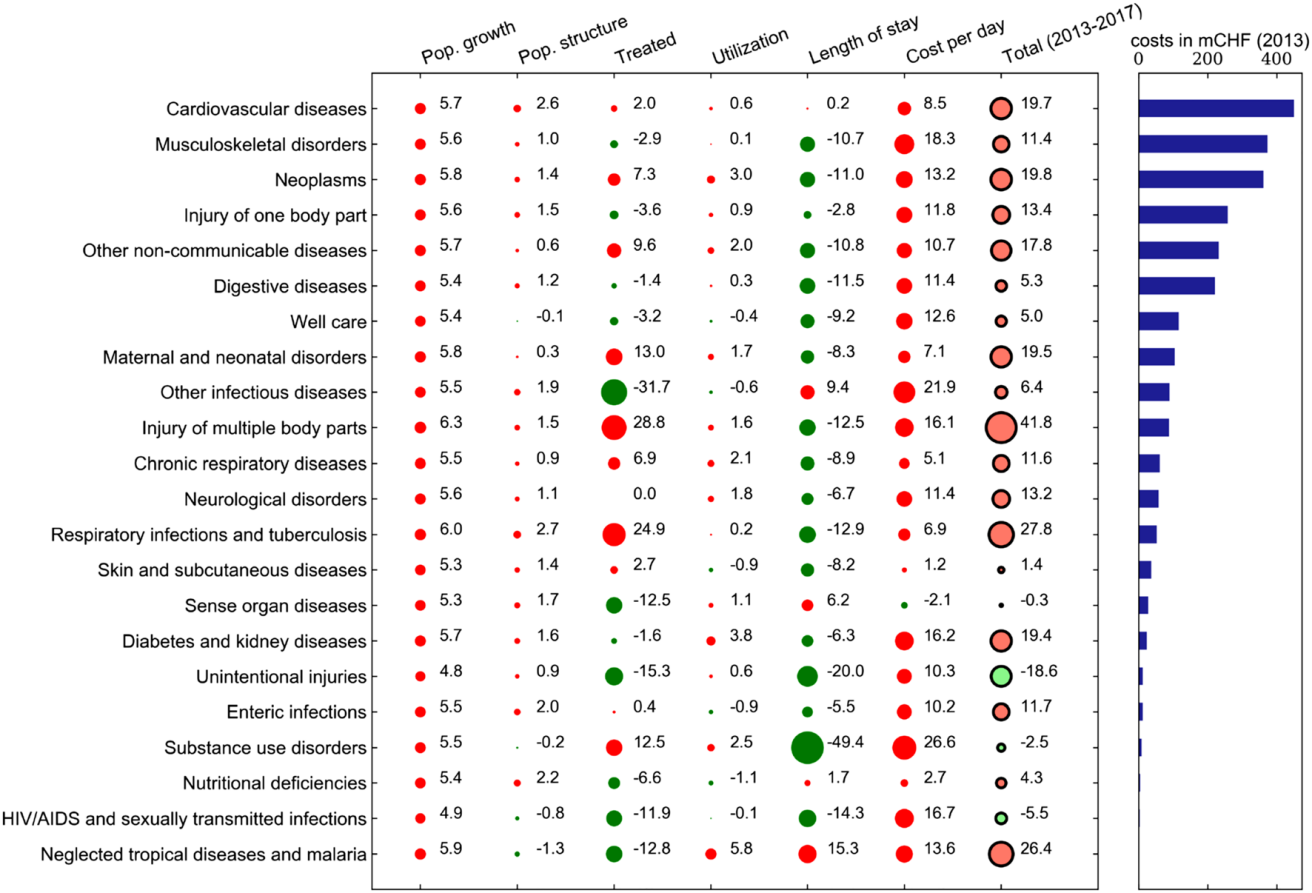
6-Factor decomposition results (2013–2017) and costs by disease in 2013 at GBD level 2

There is some heterogeneity in the effect size across diseases, as indicated by Fig. 4. In this figure, the results from the 100 disease-specific estimations are aggregated at the GBD level 2 (with 22 groups of diseases; mental disorders as well as the group of non-distinctive codes are not shown here). In the aggregation, no weighting takes place, i.e. the single factors’ contributions are summed up across all level 3 diseases within each level 2 category. The size of a circle corresponds to the magnitude of the association, while the color shows the direction of change. A red circle is associated with a cost increase, whereas a green circle is associated with a cost decrease. The last column of black-rimmed circles summarizes the total cost increase between 2013 and 2017 relative to the total costs in 2013. To put these relative changes into context, the bars on the right side of the figure show the aggregate costs for each disease in 2013. The number of treated patients as a share of each age/sex group’s population was associated with a cost increase of 7.3% for neoplasms, 24.9% for respiratory infections and tuberculosis and 28.8% for injuries of multiple body parts. The effect of this factor varied substantially across disease groups. For some communicable diseases, there was a reduction in the treated population (e.g. nutritional deficiencies and other infectious diseases), and also some expensive non-communicable diseases such as musculoskeletal disorders and digestive diseases showed small reductions associated with this factor. The utilization factor association was generally small, amounting to less than $+$ / − 1% of the total cost increase for the majority of diseases. The two most important factors associated with the cost change were length of stay and costs per day. In general, the two effects were in opposite direction, with length of stay being negatively and cost per day being positively associated with the cost change between 2013 and 2017. Only five disease groups showed a positive association of length of stay with costs, and only one disease group (sense organ diseases) showed a negative association of costs per day with costs. For very few diseases, including the most expensive disease group of cardiovascular diseases, there was a positive association of both factors with costs.

**Fig. 5:**
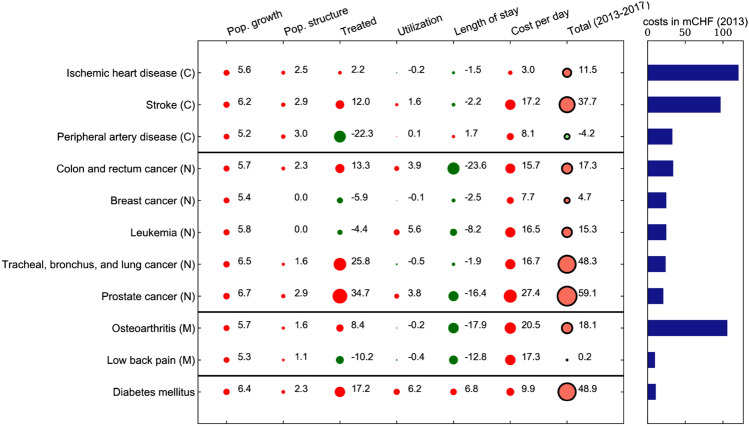
6-factor decomposition results (2013–2017) and costs by disease in 2013 for selected diseases at GBD level 3

These heterogeneous effects become even more visible if we look at a more granular disease level. Figure 5 shows the decomposition for eleven single diseases from the GBD level 2 groups cardiovascular diseases (C), neoplasms (N) and musculoskeletal disorders (M), as well as diabetes mellitus. These diseases are shown in the order of their total costs in 2013 within their GBD level 2 group. Cardiovascular diseases, neoplasms and musculoskeletal disorders were the three most expensive disease groups in both 2013 and 2017. Diabetes showed one of the strongest increase in costs (48.9%) across all diseases. Peripheral artery disease was an outlier among the cardiovascular diseases, as its aggregate costs decreased. This was mainly due to a strong negative association between the relative number of treated patients and costs. By contrast, stroke showed a strong cost increase, which was mainly due to an increase in costs per day. Within the neoplasms group, prostate cancer as well as tracheal, bronchus and lung cancer deserve special attention because of their significant cost increase (+ 59.1% and + 48.3%, respectively). Despite the reduction in length of stay for both conditions, the association between both the share of treated patients and the costs per day yielded a positive net cost change. Osteoarthritis showed an increase in the relative number of treated patients, whereas the converse held true for low back pain. For diabetes mellitus, we observe a positive association with costs for all six factors. This means that relatively more patients received an inpatient treatment, those patients had a higher utilization and longer stays with higher costs per day.

The complete results are provided in Tables 5 (level 2) and 7 (level 3) in the appendix.

The sum of the length of stay and the costs per day effect is roughly equal to the effect of costs per case. We checked this with a decomposition analysis including only five factors. The results are very similar, i.e. the costs per case effect is very close to the sum of the length of stay and the costs per day effect (see detailed results by disease in Tables 6 and 8 in the appendix). While the average costs per case across all cases increased by 5.2%, there were decreases in some disease groups such as maternal and neonatal disorders (associated with a reduction of aggregate costs by – 1.2%), chronic respiratory diseases (– 3.8%) or skin and subcutaneous diseases (– 7.1%). These diseases showed increasing costs per day, but the reduced length of stay offset this increase and yielded a negative contribution of costs per case.

### Details for cost categories

**Fig. 6:**
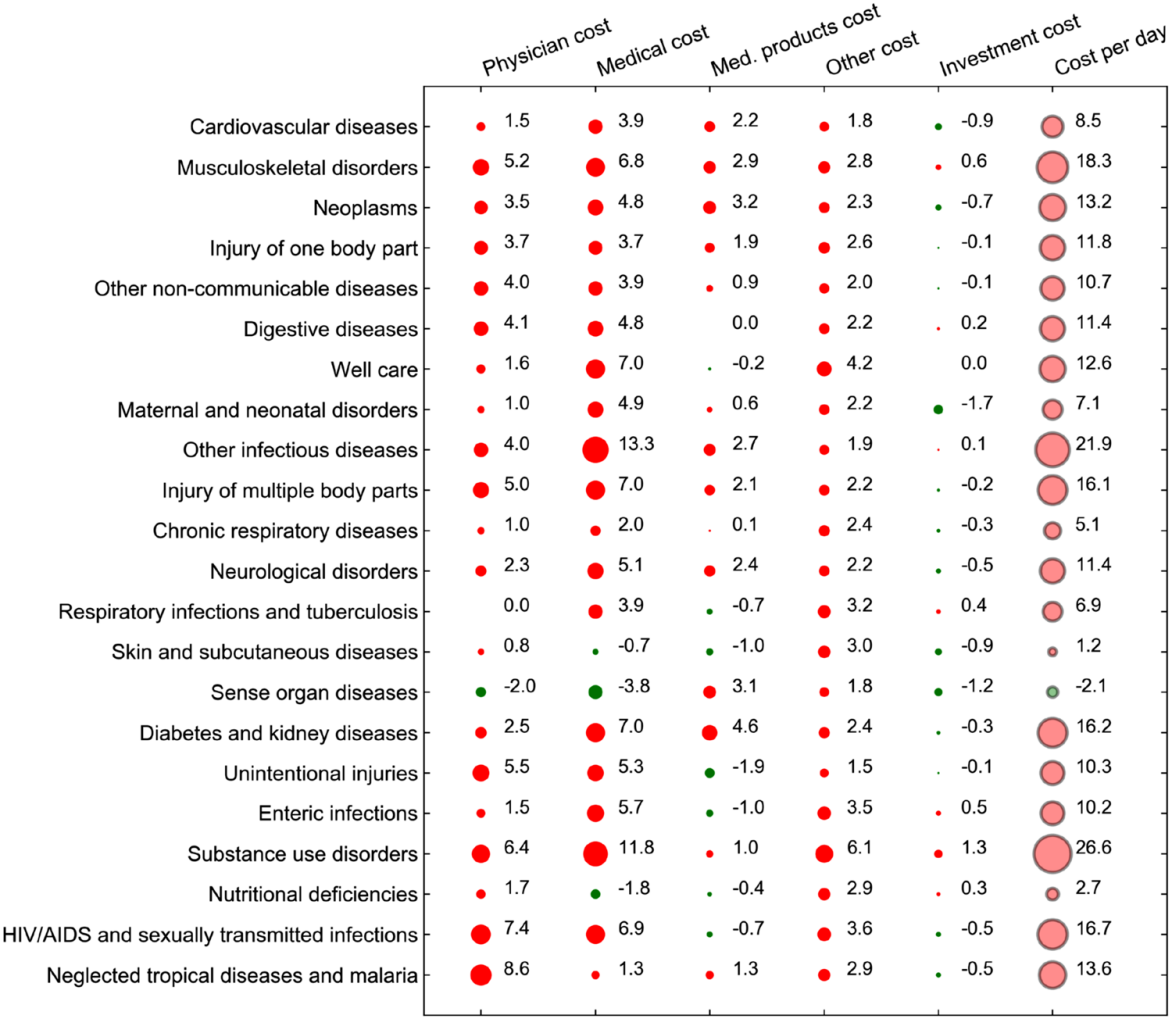
Decomposition details for cost component, GBD level 2

In this section, we explore the cost per day factor in more detail by splitting it up into the five cost categories. Figure 6 shows this decomposition result. This figure has the same structure and contains the same set of diseases as Fig. 4, but it shows the contribution of the five cost components in the first five columns and the costs per day instead of the total costs in the last column with black-rimmed circles. Also at a more granular level, the two most important cost factors are the physician and medical costs. Musculoskeletal disorders showed the strongest associations of these two categories with costs among the top three conditions (+ 5.2% and + 6.8%, respectively). Neoplasms as well as diabetes and kidney diseases had the strongest association of medical products costs (containing drugs) with aggregate costs (+ 4.6% and + 3.2%, respectively). Sense organ diseases also exhibited an increase in this cost category, but they were one of the few examples for which the physician and medical costs were negatively associated with the cost change. Overall, the medical products cost category varied substantially across diseases, with several communicable diseases showing a negative association with the total cost increase.

### Decomposition across the distribution

The costs per day and the length of stay effect for each disease represent the sum over the effects within each age/sex group. Ignoring the age/sex dimension, but instead looking at different areas of the distribution of costs per case by disease allows us to investigate whether the least or the most expensive cases contributed more to the observed changes. Each factor’s effect can be interpreted as a weighted average over the group specific effect of five sections across the cost distribution. Since we applied a slightly different specification for this analysis, the absolute values differ slightly compared to the 6-factor decomposition. The general pattern is the same. We only focus on the contribution of each distributional section (< 10th percentile, between the 10th and 25th percentile, 25th–75th percentile, 75th–90th percentile, > 90th percentile) to the total effect of each factor by disease. The contributions of each of the five sections to the total cost change by disease (at GBD level 2) are shown in the appendix (see Fig. 9). In general, the share of the 10% most expensive cases dominated the bottom of the distribution.

**Fig. 7:**
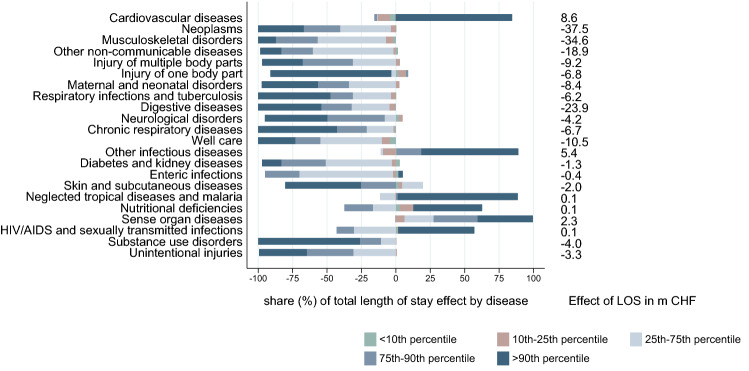
Contribution of distributional parts to total length of stay (LOS) effect by disease, GBD level2

**Fig. 8:**
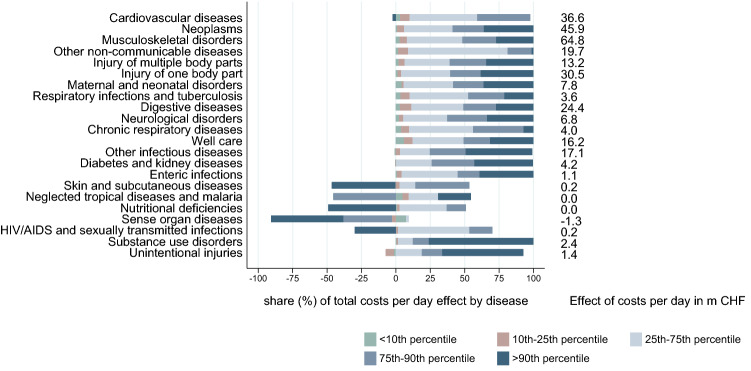
Contribution of distributional parts to total cost per day effect by disease, GBD level2

**Fig. 9:**
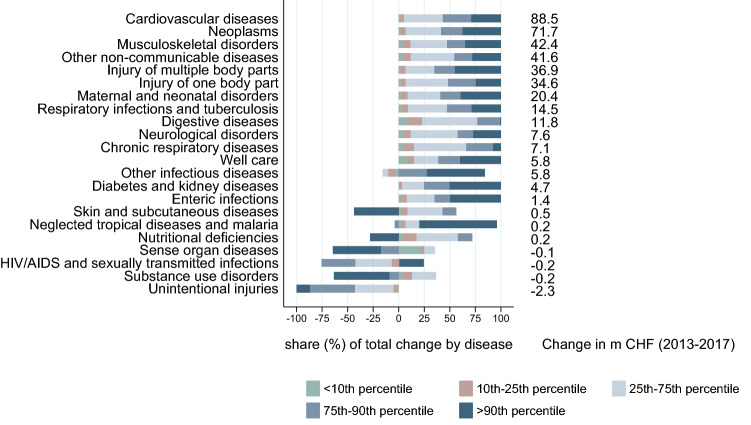
Contribution of distributional sections to total change in costs by disease, GBD level2

The factor decomposition isolated the association between the change in disease costs and both length of stay and costs per day by distributional sections. Figures 7 and 8 show the contribution of each of the five distribution sections to the total effect. Diseases are ranked based on the total absolute increase associated with this factor. The dark blue bar represents the cost increase of the 10% most expensive cases for each disease. We observe some heterogeneity in the effect of length of stay on the costs by disease. For cardiovascular diseases, the increase in the average length of stay is traced back only to the 10% most expensive cases. Similar patterns are observed for other diseases for which the length of stay increased on average^5^ (top of Fig. 7). There are more examples of diseases for which the average length of stay was not reduced for all the distribution sections, e.g. diabetes and kidney disease and neurological disorders. For diseases that showed a negative association along the whole distribution, the impact of the cases above the 75th percentile was mostly dominant.

The first decomposition specification including population structure showed a negative association between length of stay and costs per day. This was not necessarily true when looking at single sections of the distribution. There was an increase in the length of stay for the most expensive cases for other infectious diseases, but this effect was reinforced by an increase in costs per day for the same group. For most diseases, there was an increase in costs per day along the whole distribution. Noteworthy, there was a very small association of a reduction in the costs per day for the most expensive cardiovascular cases.

## Conclusion and discussion

### Summary

Given the rapid increase in health care spending in Switzerland in the last few years, it is pivotal to better understand the factors associated with this increase. This study was the first to decompose inpatient care costs by disease in Switzerland, using detailed cost data for the canton of Zurich, between 2013 and 2017. It assessed the impact of six fundamental factors on the change of disease costs over time. The most important factor was the increase in the costs per day, which was partly offset by a reduction in the average length of stay. Population growth and age and sex structure were related to about half of the increase. A minor part was associated with an increase in the number of people treated relative to the population. This study contributed to the existing literature in three important ways. First, it included one additional factor in the decomposition specification, namely length of stay. Second, it further decomposed the costs per day factor into five cost components. Third, it reported results not only for the average, but also for the full cost distribution.

### Discussion

The data we used does not reflect spending at prices charged to payers but production costs incurred by the hospitals. It is therefore a more objective measure of the resources needed to provide treatment for each disease than spending. However, the cost changes observed in this study do not necessarily correspond to the actual health care spending changes by disease, since hospitals might either realize profits or the costs by disease might not perfectly correspond to the spending for the diseases.

Although we used data at the individual level, an aggregation of the cost data at the disease and age/sex group level was appropriate for the question we aimed to answer. Decomposition methods usually used with individual level data, i.e. Oaxaca and Blinder type decompositions [33, 34], are of limited use here since they aim at explaining the mean outcome (in our case mean costs per day or case) and thus ignore the factors leading to the aggregate change such as population growth or utilization. One strength of our study lies in its ability to separate the propensity to be treated from the utilization as well as the intensity and price of treatment (captured by length of stay and costs per day).

The observed positive association between population structure and costs is due to the fact that patients in higher age groups exhibit more stays and higher costs per case. It has been shown for Switzerland that costs per patient and hospital days per person in the inpatient sector increase significantly beyond the age of 60 [36]. Since we controlled for other factors, the cost increase that is linked to a change in population structure is small.

Several reasons may explain the increase in the share of people being treated, which was associated with a cost increase of 1.9%. The first one might be supplier-induced demand. A recent study for Germany has shown that the steady increase in hospital admissions can be explained only partially by demand-side factors such as time-to-death and morbidity; about 80% is accounted for by supply-side effects resulting from the DRG reimbursement system [37]. The second reason is linked to technological progress: new diagnostic and treatment options allow more patients to be successfully treated, which possibly leads to better health outcomes [38]. The third reason is a more technical one. We measured the treated prevalence as the share of unique patients within each age/sex group. Since some patients live outside of the canton of Zurich, but this information was not accessible to us, the factor is also influenced by inter-cantonal patient migration and might thus be slightly biased. The share of the net patient migration has increased from 9.5 to 10.7% of the stays between 2013 and 2017 [39]. We can therefore not rule out that for some diseases, part of the effect of the treated factors is driven by patients coming from other cantons. A fourth explanation is the underlying disease prevalence: if more people suffer from a disease, the number of treated patients is likely to increase too. However, our study was not able to capture the health-related needs of the patients and thus ignored the fact that some diseases become more or less prevalent. The studies by Dieleman et al. [5] and Zhai et al. [23] included age/sex-specific prevalence estimates for each disease from the GBD project in their decompositions and reported utilization per prevalent case instead of per treated patient. Even though national prevalence estimates are also available for Switzerland from the GBD project, we did not include these in our analysis of sub-national data. Since we only focus on one health care sector, our two factors treated and utilization provide policy makers more easily interpretable results than utilization per (clinically) prevalent case.^6^ For some communicable diseases and injuries, we observed a reduction in the share of treated patients. This might be due to a shift from inpatient to outpatient treatments. Despite this development, the number of treated people has further increased for most diseases. This suggests that surgeries in the inpatient setting do not decrease to the same extent as the outpatient treatments increase [42].

The observed decrease in the length of stay and the increase in the utilization factor might both be linked to the implementation of the DRG reimbursement system. Hospitals have been incentivized to discharge patients early and to produce more stays (e.g. [43] for the United States). Previous research for Switzerland provided evidence that the system change led to an increase in hospital readmissions [44]. Readmissions within 18 days of treatment are, however, already included in our records and do not constitute an additional stay, i.e. the utilization factor as defined here captures the increase in stays per patient on top of that. The differentiation between the number of stays per patient and the length of stay thus proves to be relevant, as the effects go in opposite directions. Utilization defined as number of days per patient like in [5] captures both effects at the same time. Furthermore, our study confirms previous evidence for Switzerland, as it found a strong association between the reduced length of stay and costs [45]. The heterogeneity of the length of stay factor association across diseases might indicate different levels of efficiency margins for different diseases.

The reduction in length of stay was offset by an even higher increase in costs per day.^7^ This result is in line with the results by Dieleman et al. for the US, who found an increase in costs per hospital day and a reduction in bed-days per prevalent case [5]. The reason might be a compression of (more intensive) treatment into a shorter period of time. The relative size of the two competing effects of length of stay (negative) and costs per day (positive) in our study (7.3%/12.1% = 60%) is the same as the ratio of bed-days per patient and costs per day in [5]. This accordance is surprising, even though they defined the utilization term in a slightly different way than we did. The costs per day factor likely captures to some extent higher input prices, e.g. due to technological progress in drugs and higher wages, and an increase in treatment intensity, e.g. due to technological progress or supplier-induced demand. This is, however, only likely in the case that the additional treatment leads to higher reimbursement for the hospital. A significant part of the increase in costs per day is probably due to an increase in input prices for medical staff. Our costs per day decomposition showed that 8.1 of the 12.1%-points cost increase association was due to medical and physician costs, two categories mainly consisting of labor expenses. The Swiss statistics on hospitals (Krankenhausstatistik) by the Federal Statistical Office reports that the total wage bill in general hospitals in the canton of Zurich increased by 13% between 2013 and 2017 [2]. The highest increase was observed for physicians’ salaries. Their share of the total wages increased from 24.0 to 25.7%, which is equal to a rise of the wage sum for physicians by 21% in the 4 years period. A similar focus on the sources of increasing costs per inpatient case was taken previously by a study in the United States which showed that supplies and devices, room and board, intensive care and operating rooms as well as drugs contributed to more than 75% of the increase in costs per case between 2001 and 2006 [46].

Accounting for length of stay as well as costs per day is important. However, policy makers are more likely to be interested in their joint effect, i.e. costs per case. Despite a modest overall increase in average costs per case, average costs decreased for some diseases. This is surprising, as input costs, and particularly wages of hospital personnel, increased substantially over the study period.

Our analysis of distributional aspects revealed that for most diseases, the 10% most expensive cases showed the highest contribution to the factor associations. However, it also showed that the direction of the effect might differ across the distributional sections and by disease. Only recently, the literature aiming at identifying cost drivers has started to look at other measures than just the average [47, 48]. DeMeijer et al. [48] used Dutch data and showed that the impacts of various explaining variables measured at the patient level differed across the spending distribution. For hospital spending, growth was found to be largest in the middle of the distribution. In contrast to their study, we did not use an individual-level decomposition, but our results provide insights at the disease level.

### Limitations

This study has several limitations. First, we did not account for comorbidities in the allocation of costs to diseases. A recent study showed that spending estimates by disease are affected by the choice of allocation method [31]. About 19% of the cases in our data do not include any secondary diagnoses and would not be affected by a comorbidity adjustment; about 25% show six or more comorbidities. Among those cases with secondary diagnoses, the first coded secondary diagnosis was aggregated into the same disease group by our algorithm as the main diagnosis in 31% of the cases (22% for the second, 16% for the third). In such cases there is no need for a comorbidity adjustment. For a substantial part of the cases, however, there are coded comorbidities other than the main diagnosis, which would potentially require the assignment of costs to more than one disease. The second limitation of our study is the short time period. This is due to data quality and consistency across years. It would be interesting to see in the future whether similar patterns are observed over time even several years after the introduction of the DRG payment system. Third, due to data availability, the study only covers one health care sector. A comparison across outpatient and inpatient would be highly relevant for policy making. Fourth, the observed positive association of the costs per day factor with aggregate disease costs does not necessarily mean treatments were not cost-effective. Our study was not able to account for outcome quality.

### Policy implications and future research

A better understanding of the epidemiological, technological and demographic trends on health care costs may be particularly useful for a sound definition of global spending budgets currently discussed in Switzerland. Effective cost containment policies require reliable estimates of the key factors influencing costs at a very granular level such as specific diseases. The majority of the cost increase was due to only three groups that caused an increasing health and cost burden and should be monitored carefully. Furthermore, it is important to know the consequences of a change in the hospital payment system for health care utilization. Our study suggests that the number of cases per treated (in)patient within each disease group is increasing, which would somehow conflict with the goal to increase efficiency in hospitals.

Future research should include other health care sectors to investigate whether there has been a shift to outpatient treatments and if yes to what extent and for which diseases. It is further important to research the reasons for the factor associations we found, e.g. the increased number of treated inpatients relative to the population and the increased number of stays per (in)patient over time. In addition, it would be interesting to assess whether the additional health care use is due to increased care needs or due to hospital-induced demand. Accounting for the cost impact of comorbidities is a very important and demanding task for future research. Some regression-based approaches have recently been proposed by the literature to adjust for concurrent diseases at the patient or the encounter level [49, 50]. Our framework could also be used to assess the impact of lower length of stay on treatment quality at the level of specific diseases. The reduction in length of stay is only desirable if there are no “bloody exits” associated with worse health outcomes. Finally, expanding the analysis to more than just average effect has shown how important it is to have a closer look at which cases contributed most to the cost increase. It is therefore vital to include distributional aspects into future cost-of-illness studies and other research looking at drivers of health care spending.

## Appendix

See Tables 2, 3, 4, 5, 6, 7, 8 and Fig. 9.

**Table 2:** Cost types included in cost categories

Cost category	Cost types included
Physician costs	Attending physicians^a^
	Hospital physicians^a^
	Physicians
	Physician reports^a^
Medical costs	Anesthetics
	Imaging
	Diagnostics
	Dialysis
	Occupational therapy
	Delivery room
	Intensive care
	Laboratory
	Logopedia
	Emergency department
	Nuclear medicine
	Operating room
	Pathology
	Nursing
	Physiotherapy
	Psychology
Medical products costs	Drugs^a^
	Blood products^a^
	Implants^a^
	Medical material^a^
	Other medical products^a^
Other costs	Primary transport^a^
	Secondary transport
	Housing
	Housing (food)
	Housing (service)
	Housing (room)
	Patient administration
	Other fixed costs
	Other case-specific costs^a^
Investment costs	Capital costs (depreciation)

^a^Direct costs (i.e. cost items directly allocated at the case level)

**Table 3:** Modified disease classification with three disease levels according to the Global Burden of Disease study

GBD Level 1	GBD Level 2	GBD Level 3
Communicable diseases	HIV/AIDS and sexually transmitted infections	
	Respiratory infections and tuberculosis	
	enteric infections	
	neglected tropical diseases and malaria	
	other infectious diseases	
	maternal and neonatal disorders	
	nutritional deficiencies	
Non-communicable diseases	Neoplasms	Lip and oral cavity cancer
		Nasopharynx cancer
		Other pharynx cancer
		Esophageal cancer
		Stomach cancer
		Colon and rectum cancer
		Liver cancer
		Gallbladder and biliary tract cancer
		Pancreatic cancer
		Larynx cancer
		Tracheal, bronchus, and lung cancer
		Malignant skin melanoma
		Non-melanoma skin cancer
		Breast cancer
		Cervical cancer
		Uterine cancer
		Ovarian cancer
		Prostate cancer
		Testicular cancer
		Kidney cancer
		Bladder cancer
		Brain and nervous system cancer
		Thyroid cancer
		Mesothelioma
		Hodgkin lymphoma
		Non-Hodgkin lymphoma
		Multiple myeloma
		Leukemia
		Other malignant neoplasms
		Other neoplasms
	Cardiovascular diseases	Rheumatic heart disease
		Ischemic heart disease
		Stroke
		Hypertensive heart disease
		Non-rheumatic valvular heart disease
		Cardiomyopathy and myocarditis
		Atrial fibrillation and flutter
		Aortic aneurysm
		Peripheral artery disease
		Endocarditis
		Other cardiovascular and circulatory diseases
	Chronic respiratory diseases	Chronic obstructive pulmonary disease
		Pneumoconiosis
		Asthma
		Interstitial lung disease and pulmonary sarcoidosis
		Other chronic respiratory diseases
	Digestive diseases	Cirrhosis and other chronic liver diseases
		Upper digestive system diseases
		Appendicitis
		Paralytic ileus and intestinal obstruction
		Inguinal, femoral, and abdominal hernia
		Inflammatory bowel disease
		Vascular intestinal disorders
		Gallbladder and biliary diseases
		Pancreatitis
		Other digestive diseases
	Neurological disorders	Alzheimer’s disease and other dementias
		Parkinson’s disease
		Epilepsy
		Multiple sclerosis
		Motor neuron disease
		Headache disorders
		Other neurological disorders
	Mental disorders	
	Substance use disorders	Alcohol use disorders
		Drug use disorders
	Diabetes and chronic kidney diseases	Diabetes mellitus
		Chronic kidney disease
		Acute glomerulonephritis
	Skin and subcutaneous diseases	
	Sense organ diseases	Blindness and vision impairment
		Age-related and other hearing loss
		Other sense organ diseases
	Musuloskeletal disorders	Rheumatoid arthritis
		Osteoarthritis
		Low back pain
		Neck pain
		Gout
		Other musculoskeletal disorders
	Other non-communicable diseases	Congenital birth defects
		Urinary diseases and male infertility
		Gynecological diseases
		Hemoglobinopathies and hemolytic anemias
		Endocrine, metabolic, blood, and immune disorders
		Oral disorders
		Sudden infant death syndrome
Injuries	Transport injuries	
	Unintentional injuries	
	Self-harm and interpersonal violence	
	Injury of one body part	
	Injury of multiple body parts	
Well care		
Non-distinctive codes

**Table 4:** Summary statistics by disease (GBD level 2) and year

	2013									2017								
Disease (GBD level 2)	Total costs per case (CHF)	Share of direct costs	Physician costs per case (CHF)	Medical products costs per case (CHF)	Medical costs per case (CHF)	Other costs per case (CHF)	Investment costs per case (CHF)	Length of stay (d)	Number of cases	Total costs per case (CHF)	Share of direct costs	Physician costs per case (CHF)	Medical products costs per case (CHF)	Medical costs per case (CHF)	Other costs per case (CHF)	Investment costs per case (CHF)	Length of stay (d)	Number of cases
HIV/AIDS and sexually transmitted infections	10735	14.5%	1977	1122	5808	873	956	5.7	268	12240	13.6%	2666	1043	6432	1258	841	6.1	222
Respiratory infections and tuberculosis	10104	10.1%	2057	752	5569	976	750	6.5	5160	9675	9.3%	1853	620	5349	1147	706	5.9	6886
Enteric infections	6391	10.6%	1398	448	3292	752	500	4.6	1823	6757	8.7%	1422	347	3548	932	508	4.4	1926
Neglected tropical diseases and malaria	12433	13.3%	2543	1405	6537	979	968	6.2	72	14142	14.5%	3139	1565	7143	1369	927	6.8	80
Other infectious diseases	21411	12.3%	3655	2218	12455	1490	1592	10.4	4193	27234	12.7%	4704	2893	15988	1980	1669	10.8	3508
Maternal and neonatal disorders	8750	8.8%	1663	341	5172	763	810	5.7	11956	8711	9.2%	1627	359	5215	881	628	5.3	14348
Nutritional deficiencies	9949	15.4%	2122	842	5166	1038	781	6.9	383	10189	14.0%	2327	793	4940	1318	811	7	390
Neoplasms	18832	21.5%	4106	2348	9605	1255	1518	7.9	19245	19303	23.2%	4368	2657	9481	1531	1267	7.2	22488
Cardiovascular diseases	19651	28.0%	3884	4424	8831	1123	1389	6.4	22892	21337	28.7%	4186	4870	9592	1463	1226	6.5	25232
Chronic respiratory diseases	11326	22.4%	2843	1001	5754	871	857	5	5362	10883	24.4%	2737	922	5437	1036	751	4.6	6229
Digestive diseases	12044	18.0%	2597	1417	6212	876	942	5.6	18338	12036	17.8%	2782	1268	6095	1028	863	5	19325
Neurological disorders	14469	13.0%	3280	1071	7667	1325	1126	7.3	3984	14993	13.9%	3355	1343	7829	1496	970	6.7	4352
Mental disorders	8230	11.6%	1865	453	4331	947	635	5.6	3349	9379	12.5%	2028	574	4899	1228	650	5.6	3594
Substance use disorders	8361	9.1%	1592	468	4536	1081	684	7.4	1072	7040	8.7%	1385	307	3784	1026	538	4.9	1242
Diabetes and kidney diseases	15129	13.9%	2649	2253	7480	1692	1054	7.9	1607	16490	13.3%	2915	2436	8271	1881	985	7.9	1760
Skin and subcutaneous diseases	10218	11.4%	2224	732	5473	951	838	6.4	3583	9725	11.1%	2160	592	5087	1183	701	6.1	3818
Sense organ diseases	7049	24.9%	1839	1276	2833	528	573	2.2	3967	7312	27.3%	1848	1516	2733	695	521	2.4	3811
Musculoskeletal disorders	13984	28.2%	3348	2635	5849	1089	1063	6.2	26654	15286	29.1%	3757	2816	6279	1380	1054	5.7	27157
Other non-communicable diseases	12104	20.2%	2755	1627	5971	834	917	4.8	19265	11990	21.6%	2879	1508	5814	976	814	4.4	22916
Unintentional injuries	14850	22.2%	3271	2884	6330	1238	1128	6.9	839	12513	19.2%	3128	1929	5486	1129	841	5.3	811
Injury of one body part	11601	17.1%	2570	1210	6002	905	914	5.4	22209	12645	17.6%	2917	1390	6276	1182	879	5.2	23112
Injury of multiple body parts	16497	22.6%	3117	2980	7964	1234	1202	6.8	5359	17139	23.2%	3503	2992	8161	1416	1067	6.3	7314
Well care	5202	7.4%	1107	219	2864	538	473	4.3	22243	5458	7.1%	1115	195	3006	705	438	3.9	22262
Non-distinctive codes	9983	15.5%	2053	1345	4968	905	712	5.9	4615	8793	13.6%	1910	1104	4191	971	616	4.6	4653
Total	12804	18.7%	2755	1785	6315	959	991	5.9	208438	13466	19.3%	2959	1893	6532	1183	899	5.5	227436

**Table 5:** Detailed 6-factor decomposition results for GBD level 2 diseases

Disease	Pop. growth (%)	Pop. structure (%)	Treated (%)	Utilization (%)	Length of stay (%)	Cost per day (%)	Total (2013–2017, %)	Disease costs (2013, mCHF)
Cardiovascular diseases	5.7	2.6	2	0.6	0.2	8.5	19.7	450
Musculoskeletal disorders	5.6	1	$-$ 2.9	0.1	$-$ 10.7	18.3	11.4	373
Neoplasms	5.8	1.4	7.3	3	$-$ 11	13.2	19.8	362
Injury of one body part	5.6	1.5	$-$ 3.6	0.9	$-$ 2.8	11.8	13.4	258
Other non$-$communicable diseases	5.7	0.6	9.6	2	$-$ 10.8	10.7	17.8	233
Digestive diseases	5.4	1.2	$-$ 1.4	0.3	$-$ 11.5	11.4	5.3	221
Well care	5.4	$-$ 0.1	$-$ 3.2	$-$0.4	$-$ 9.2	12.6	5	116
Maternal and neonatal disorders	5.8	0.3	13	1.7	$-$ 8.3	7.1	19.5	105
Other infectious diseases	5.5	1.9	$-$ 31.7	$-$ 0.6	9.4	21.9	6.4	90
Injury of multiple body parts	6.3	1.5	28.8	1.6	$-$ 12.5	16.1	41.8	88
Chronic respiratory diseases	5.5	0.9	6.9	2.1	$-$ 8.9	5.1	11.6	61
Neurological disorders	5.6	1.1	0	1.8	$-$ 6.7	11.4	13.2	58
Respiratory infections and tuberculosis	6	2.7	24.9	0.2	$-$ 12.9	6.9	27.8	52
Non$-$distinctive codes	5	1.9	$-$ 10.2	0.2	$-$ 19.1	10.9	$-$ 11.2	46
Skin and subcutaneous diseases	5.3	1.4	2.7	$-$ 0.9	$-$ 8.2	1.2	1.4	37
Sense organ diseases	5.3	1.7	$-$ 12.5	1.1	6.2	$-$ 2.1	$-$ 0.3	28
Mental disorders	5.8	2.1	0.6	2.2	$-$ 3.1	14.6	22.3	28
Diabetes and kidney diseases	5.7	1.6	$-$ 1.6	3.8	$-$ 6.3	16.2	19.4	24
Unintentional injuries	4.8	0.9	$-$ 15.3	0.6	$-$ 20	10.3	$-$ 18.6	12
Enteric infections	5.5	2	0.4	$-$ 0.9	$-$ 5.5	10.2	11.7	12
Substance use disorders	5.5	$-$ 0.2	12.5	2.5	$-$ 49.4	26.6	$-$ 2.5	9
Nutritional deficiencies	5.4	2.2	$-$ 6.6	$-$ 1.1	1.7	2.7	4.3	4
HIV/AIDS and sexually transmitted infections	4.9	$-$ 0.8	$-$ 11.9	$-$ 0.1	$-$ 14.3	16.7	$-$ 5.5	3
Neglected tropical diseases and malaria	5.9	$-$ 1.3	$-$ 12.8	5.8	15.3	13.6	26.4	1

**Table 6:** Detailed 5-factor decomposition results for GBD level 2 diseases

Disease	Pop. growth (%)	Pop. structure (%)	Treated (%)	Utilization (%)	Costs per case (%)	Total (2013–2017, %)	Disease costs (2013, mCHF)
Cardiovascular diseases	5.7	2.6	1.9	0.5	8.9	19.7	450
Musculoskeletal disorders	5.6	1	$-$ 2.9	0.1	7.6	11.4	373
Neoplasms	5.8	1.4	7.2	3	2.4	19.8	362
Injury of one body part	5.6	1.5	$-$ 3.6	0.9	9	13.4	258
Other non$-$communicable diseases	5.7	0.6	9.7	2	$-$ 0.1	17.8	233
Digestive diseases	5.4	1.2	$-$ 1.4	0.3	$-$ 0.1	5.3	221
Well care	5.4	$-$ 0.1	$-$ 3.2	$-$ 0.4	3.3	5	116
Maternal and neonatal disorders	5.8	0.3	13	1.7	$-$ 1.2	19.5	105
Other infectious diseases	5.5	2	$-$ 32	$-$ 0.6	31.6	6.4	90
Injury of multiple body parts	6.3	1.5	28.8	1.5	3.7	41.8	88
Chronic respiratory diseases	5.5	0.9	6.9	2.1	$-$3.8	11.6	61
Neurological disorders	5.6	1.1	$-$ 0.1	1.8	4.8	13.2	58
Respiratory infections and tuberculosis	6	2.7	24.9	0.2	$-$ 6	27.8	52
Non$-$distinctive codes	5	2	$-$ 9.8	0.2	$-$ 8.5	$-$ 11.2	46
Skin and subcutaneous diseases	5.3	1.4	2.7	$-$ 0.9	$-$ 7.1	1.4	37
Sense organ diseases	5.3	1.7	$-$ 12.4	1.1	4	$-$ 0.3	28
Mental disorders	5.8	2.1	0.6	2.2	11.6	22.3	28
Diabetes and kidney diseases	5.7	1.6	$-$ 1.3	3.9	9.5	19.4	24
Unintentional injuries	4.8	0.9	$-$ 15.2	0.7	$-$ 9.7	$-$ 18.6	12
Enteric infections	5.5	2	0.5	$-$ 0.9	4.6	11.7	12
Substance use disorders	5.2	$-$ 0.1	10.8	2.5	$-$ 20.9	$-$ 2.5	9
Nutritional deficiencies	5.3	2.2	$-$ 6.8	$-$ 1.4	4.9	4.3	4
HIV/AIDS and sexually transmitted infections	4.9	$-$ 0.9	$-$ 10.9	0.4	0.9	$-$ 5.5	3
Neglected tropical diseases and malaria	5.9	$-$ 1.3	$-$ 11.4	6.1	27	26.4	1

**Table 7:** Detailed 6-factor decomposition results for GBD level 3 diseases

Classification	Disease	Pop. growth (%)	Pop. structure (%)	Treated (%)	Utilization (%)	Length of stay (%)	Cost per day (%)	Total (2013–2017, %)	Disease costs (2013, mCHF)
Cardio	Other cardiovascular and circulatory diseases	5.9	2.7	$-$ 1.8	1.7	5.1	11	24.6	125.3
Cardio	Ischemic heart disease	5.6	2.5	2.2	$-$ 0.2	$-$ 1.5	3	11.5	121.1
Cardio	Stroke	6.2	2.9	12	1.6	$-$ 2.2	17.2	37.7	96.7
Cardio	Peripheral artery disease	5.2	3	$-$ 22.3	0.1	1.7	8.1	$-$ 4.2	32.6
Cardio	Aortic aneurysm	5.6	2.4	1.3	$-$ 1.2	20.1	$-$ 15.7	12.4	28.5
Cardio	Atrial fibrillation and flutter	7.4	3.1	51	3	$-$15.5	34.3	83.2	16.2
Cardio	Cardiomyopathy and myocarditis	4.5	0.2	4.8	$-$ 7.4	$-$ 25.8	$-$ 1.4	$-$ 25	12.4
Cardio	Rheumatic heart disease	2.9	1.5	$-$ 60	$-$ 0.1	$-$ 14.1	$-$ 8.5	$-$ 78.3	9.6
Cardio	Hypertensive heart disease	4.8	2.8	$-$ 17.4	$-$ 1	$-$ 28.6	19.1	$-$ 20.2	3
Cardio	Endocarditis	6.1	3.1	31.2	0	3.2	18.4	62.1	4.5
Musculoskeletal	Other musculoskeletal disorders	5.5	0.8	$-$ 7.3	0.3	$-$ 7.7	17.6	9.2	254.8
Musculoskeletal	Osteoarthritis	5.7	1.6	8.4	$-$ 0.2	$-$ 17.9	20.5	18.1	105.8
Musculoskeletal	Low back pain	5.3	1.1	$-$ 10.2	$-$ 0.4	$-$ 12.8	17.3	0.2	9.6
Musculoskeletal	Rheumatoid arthritis	4.3	0.6	$-$ 14.9	$-$ 4.3	$-$ 17.4	$-$ 1.1	$-$ 32.8	1
Musculoskeletal	Gout	5.2	3.7	$-$ 7	$-$ 0.5	$-$ 2	2.6	2.1	1.5
Neoplasms	Other neoplasms	5.5	1	1.9	1.5	$-$ 12.9	13.1	10.2	62
Neoplasms	Colon and rectum cancer	5.7	2.3	13.3	3.9	$-$ 23.6	15.7	17.3	34.5
Neoplasms	Other malignant neoplasms	5.3	0.7	2	3.7	$-$ 22.8	7.3	$-$ 3.9	29.7
Neoplasms	Breast cancer	5.4	0	$-$ 5.9	$-$ 0.1	$-$ 2.5	7.7	4.7	24.6
Neoplasms	Leukemia	5.8	0	$-$ 4.4	5.6	$-$ 8.2	16.5	15.3	24.5
Neoplasms	Tracheal, bronchus, and lung cancer	6.5	1.6	25.8	$-$ 0.5	$-$ 1.9	16.7	48.3	24.2
Neoplasms	Prostate cancer	6.7	2.9	34.7	3.8	$-$ 16.4	27.4	59.1	20.6
Neoplasms	Bladder cancer	6.2	3.8	18.1	2.5	$-$ 6.1	14.8	39.4	15
Neoplasms	Non$-$Hodgkin lymphoma	6.2	1	3.9	11.9	$-$ 4.3	13.1	31.9	14
Neoplasms	Brain and nervous system cancer	5.6	1.1	11.3	4.3	$-$ 14.5	1.7	9.5	12.7
Neoplasms	Pancreatic cancer	5.8	1.9	17.7	7.6	$-$ 16.7	7.8	24.1	11.4
Neoplasms	Ovarian cancer	5.2	0.3	5.7	$-$ 1.7	$-$14.3	4.2	$-$ 0.6	11.3
Neoplasms	Esophageal cancer	4.9	1.9	$-$ 23.2	0.1	$-$ 13.2	16.9	$-$ 12.5	10.7
Neoplasms	Stomach cancer	6.5	1.9	31.5	$-$ 9.5	$-$ 5.8	18	42.5	6.9
Neoplasms	Lip and oral cavity cancer	6.2	1.8	4.4	2.8	11.6	9.6	36.4	6.8
Neoplasms	Liver cancer	6.6	2.6	22.7	9.1	$-$ 0.4	15.4	56	6.7
Neoplasms	Multiple myeloma	5.5	1.1	4.4	2.7	$-$ 15.9	15.9	13.8	6.4
Neoplasms	Kidney cancer	6.5	2.9	29	5.6	$-$ 14.9	17.1	46.2	6.3
Neoplasms	Uterine cancer	5.4	0.5	$-$ 1.4	8.7	$-$ 17.5	10.2	5.9	5.2
Neoplasms	Non$-$melanoma skin cancer	5.4	3.8	4.6	$-$ 9.2	$-$ 1.1	1.2	4.7	4.6
Neoplasms	Malignant skin melanoma	5.9	1.7	3.8	4.8	8.6	4.7	29.5	4.2
Neoplasms	Other pharynx cancer	5.4	2	$-$ 37.6	4.3	11.3	17.1	2.6	3.5
Neoplasms	Cervical cancer	5.9	0.2	$-$ 4	6.4	$-$ 3.6	17.1	21.9	3.2
Neoplasms	Thyroid cancer	6.4	0.6	5.7	8	$-$ 16.8	34.6	38.4	3
Neoplasms	Gallbladder and biliary tract cancer	6	3.3	$-$ 9.6	15.9	$-$ 2.1	16.3	29.7	3
Neoplasms	Hodgkin lymphoma	5	$-$ 0.6	$-$ 2.4	3.7	24.7	$-$3.2	27.2	1.5
Neoplasms	Larynx cancer	5.5	2.6	$-$ 18.7	12.7	19	$-$ 5.9	15.2	1.5
Neoplasms	Testicular cancer	6.2	$-$ 1.3	4.4	9.2	10.5	13.5	42.6	1.2
Neoplasms	Nasopharynx cancer	2.7	1.1	$-$ 11.2	2.4	$-$ 6.2	$-$ 9.4	$-$ 20.5	0.4
Neoplasms	Mesothelioma	5.6	1.5	$-$ 3.9	$-$ 3.8	$-$ 11.2	19.3	7.5	2.8
OtherNCD	Endocrine, metabolic, blood, and immune disorders	5.5	0.4	15.5	1.1	$-$ 21.6	9.3	10.2	64.8
OtherNCD	Urinary diseases and male infertility	6.3	2.2	26.8	1.2	$-$ 4.6	11.1	43.1	59.4
OtherNCD	Congenital birth defects	5.5	0.7	$-$ 6.7	5.9	$-$ 1.9	5.9	9.3	52.5
OtherNCD	Gynecological diseases	5.3	$-$ 1	$-$ 7	$-$ 0.5	$-$ 14.1	17.8	0.6	43.8
OtherNCD	Oral disorders	6.5	$-$ 1.7	36.7	2.9	$-$ 8.3	15.2	51.3	7.9
OtherNCD	Hemoglobinopathies and hemolytic anemias	5.4	1.1	1.5	3.3	$-$ 13.3	4.5	2.6	4.9
Digestive	Other digestive diseases	5.3	1.3	$-$ 5.3	$-$ 0.4	$-$ 12.9	12.5	0.5	61.4
Digestive	Inguinal, femoral, and abdominal hernia	5.6	1.4	6.2	0	$-$ 7.6	7.6	13.2	34
Digestive	Gallbladder and biliary diseases	5.4	1.5	1.5	$-$ 1.7	$-$ 14.5	13.2	5.4	33.7
Digestive	Appendicitis	5.5	$-$ 0.4	$-$ 1.6	$-$ 1.2	$-$ 12.4	17	7	18.8
Digestive	Cirrhosis and other chronic liver diseases	5.2	0.6	$-$ 12.2	4.7	$-$ 6.3	4.4	$-$ 3.7	18.8
Digestive	Paralytic ileus and intestinal obstruction	4.9	2.1	$-$ 15	1.8	$-$ 15.1	10.1	$-$ 11.1	17.5
Digestive	Upper digestive system diseases	5.5	1.6	$-$ 1.7	1.1	$-$ 8.2	10.5	8.8	14.9
Digestive	Pancreatitis	5.8	0.8	17.6	$-$ 1	$-$ 5.2	9.9	27.9	9.9
Digestive	Inflammatory bowel disease	5.5	0.2	$-$10.6	5	$-$ 0.9	12	11.1	7.9
Digestive	Vascular intestinal disorders	6.7	2.5	53.6	0.4	$-$ 52.7	27.1	37.6	4.1
Chronic respiratory	Other chronic respiratory diseases	5.6	$-$ 0.1	12.2	$-$ 0.4	$-$ 10.6	5.7	12.4	35.7
Chronic respiratory	Chronic obstructive pulmonary disease	5.4	2.4	$-$ 3.6	4.7	$-$ 4.9	3	6.9	19.7
Chronic respiratory	Interstitial lung disease and pulmonary sarcoidosis	5.7	2.5	19.8	10.7	$-$ 18	5.9	26.7	3.9
Chronic respiratory	Asthma	5.8	1.1	$-$ 16.1	5.8	4.5	20.1	21.2	1.3
Chronic respiratory	Pneumoconiosis	1.9	0.9	$-$ 6.7	$-$ 6.7	$-$ 6.7	$-$ 6.7	$-$ 24	0.1
Neurological	Epilepsy	5.5	0	3.8	2.1	$-$ 18.5	15.8	8.8	21.5
Neurological	Other neurological disorders	5.9	1.1	$-$ 1.4	1.5	8.6	9.2	24.9	21.4
Neurological	Alzheimer’s disease and other dementias	5.3	3.3	4.4	2.1	$-$ 24.4	10.6	1.3	6.5
Neurological	Parkinson’s disease	5.2	2.7	$-$ 3.9	2.7	$-$ 0.9	6.4	2.3	5.6
Neurological	Multiple sclerosis	4.5	0.7	$-$ 12	$-$ 7	$-$ 3	$-$ 1.8	$-$ 18.6	1
Neurological	Motor neuron disease	4.7	2.6	$-$ 25.6	3.7	$-$ 8.9	6.3	$-$ 17.1	0.9
Neurological	Headache disorders	6.7	$-$ 0.4	34.6	3.6	1.6	14	60	0.8
Skin and subcutaneous	Skin and subcutaneous diseases	5.3	1.4	2.7	$-$ 0.9	$-$ 8.2	1.2	1.4	36.6
Sense organ	Blindness and vision impairment	5.4	2	$-$ 14.6	2.8	7.6	0	3.1	18.2
Sense organ	Other sense organ diseases	5.1	1.2	$-$ 8.6	$-$ 2	3.6	$-$ 6.2	$-$ 6.7	9.8
Diabetes	Chronic kidney disease	5	1	$-$18.8	1.3	$-$ 18.4	21.7	$-$ 8.2	12.8
Diabetes	Diabetes mellitus	6.4	2.3	17.2	6.2	6.8	9.9	48.9	11.4
Diabetes	Acute glomerulonephritis	3.9	1.8	44.1	44.1	44.1	44.1	182	0.1
Substance use	Alcohol use disorders	5.4	0.4	5	4.8	$-$ 21	11.5	6.2	6
Substance use	Drug use disorders	5.7	$-$ 1.2	27.3	$-$ 2	$-$ 106	56.8	$-$ 19.6	3
Mental	Mental disorders	5.8	2.1	0.6	2.2	$-$ 3.1	14.6	22.3	27.6
Communicable	maternal and neonatal disorders	5.8	0.3	13	1.7	$-$ 8.3	7.1	19.5	104.6
Communicable	other infectious diseases	5.5	1.9	$-$ 31.7	$-$ 0.6	9.4	21.9	6.4	89.8
Communicable	Respiratory infections and tuberculosis	6	2.7	24.9	0.2	$-$ 12.9	6.9	27.8	52.1
Communicable	enteric infections	5.5	2	0.4	$-$ 0.9	$-$ 5.5	10.2	11.7	11.7
Communicable	nutritional deficiencies	5.4	2.2	$-$ 6.6	$-$ 1.1	1.7	2.7	4.3	3.8
Communicable	HIV/AIDS and sexually transmitted infections	4.9	$-$ 0.8	$-$ 11.9	$-$ 0.1	$-$ 14.3	16.7	$-$ 5.5	2.9
Communicable	neglected tropical diseases and malaria	5.9	$-$ 1.3	$-$ 12.8	5.8	15.3	13.6	26.4	0.9
Injuries	Injury of one body part	5.6	1.5	$-$ 3.6	0.9	$-$ 2.8	11.8	13.4	257.7
Injuries	Injury of multiple body parts	6.3	1.5	28.8	1.6	$-$ 12.5	16.1	41.8	88.4
Injuries	Unintentional injuries	4.8	0.9	$-$ 15.3	0.6	$-$ 20	10.3	$-$ 18.6	12.5
Well care	Well care	5.4	$-$ 0.1	$-$ 3.2	$-$ 0.4	$-$ 9.2	12.6	5	115.7
Non-distinctive	Non$-$distinctive codes	5	1.9	$-$ 10.2	0.2	$-$ 19.1	10.9	$-$ 11.2	46.1

**Table 8:** Detailed 5-factor decomposition results for GBD level 3 diseases

Classification	Disease	Pop. growth (%)	Pop. structure (%)	Treated (%)	Utilization (%)	Costs per case (%)	Total (2013–2017, %)	Disease costs (2013, mCHF)
Cardio	Other cardiovascular and circulatory diseases	5.9	2.7	$-$1.8	1.7	16.2	24.6	125.3
Cardio	Ischemic heart disease	5.6	2.5	2.2	$-$0.2	1.5	11.5	121.1
Cardio	Stroke	6.2	2.9	12	1.7	15	37.7	96.7
Cardio	Peripheral artery disease	5.2	3	$-$22.3	0	9.9	$-$4.2	32.6
Cardio	Aortic aneurysm	5.5	2.4	1.1	$-$1.4	4.8	12.4	28.5
Cardio	Atrial fibrillation and flutter	7.3	3.1	50.1	3.1	19.5	83.2	16.2
Cardio	Cardiomyopathy and myocarditis	4.6	0.2	4.7	$-$7.9	$-$26.6	$-$25	12.4
Cardio	Rheumatic heart disease	2.9	1.5	$-$60.1	$-$0.6	$-$21.9	$-$78.3	9.6
Cardio	Endocarditis	6	2.7	25.8	$-$1.1	28.8	62.1	4.5
Cardio	Hypertensive heart disease	4.7	2.8	$-$17.4	$-$0.9	$-$9.5	$-$20.2	3
Musculoskeletal	Other musculoskeletal disorders	5.5	0.8	$-$7.2	0.3	9.9	9.2	254.8
Musculoskeletal	Osteoarthritis	5.7	1.6	8.4	$-$0.1	2.6	18.1	105.8
Musculoskeletal	Low back pain	5.3	1.1	$-$10	$-$0.4	4.4	0.2	9.6
Musculoskeletal	Gout	5.2	3.8	$-$6.3	$-$0.3	$-$0.4	2.1	1.5
Musculoskeletal	Rheumatoid arthritis	4.3	0.5	$-$13.4	$-$4.5	$-$19.7	$-$32.8	1
Neoplasms	Other neoplasms	5.5	1	1.9	1.5	0.3	10.2	62
Neoplasms	Colon and rectum cancer	5.7	2.3	13.2	4	$-$7.8	17.3	34.5
Neoplasms	Other malignant neoplasms	5.2	0.6	2	3.6	$-$15.3	$-$3.9	29.7
Neoplasms	Breast cancer	5.4	0	$-$5.9	$-$0.1	5.2	4.7	24.6
Neoplasms	Leukemia	5.8	0	$-$5	5.5	9	15.3	24.5
Neoplasms	Tracheal, bronchus, and lung cancer	6.5	1.6	25.8	$-$0.5	14.9	48.3	24.2
Neoplasms	Prostate cancer	6.7	2.9	34.6	3.8	11.1	59.1	20.6
Neoplasms	Bladder cancer	6.2	3.8	18.1	2.5	8.8	39.4	15
Neoplasms	Non$-$Hodgkin lymphoma	6.2	1	3	10.6	11.1	31.9	14
Neoplasms	Brain and nervous system cancer	5.6	1.1	11.3	4.3	$-$12.8	9.5	12.7
Neoplasms	Pancreatic cancer	5.8	1.9	17.8	7.7	$-$9.1	24.1	11.4
Neoplasms	Ovarian cancer	5.2	0.3	5.7	$-$1.8	$-$10.1	$-$0.6	11.3
Neoplasms	Esophageal cancer	4.9	1.9	$-$23.3	0.1	3.8	$-$12.5	10.7
Neoplasms	Stomach cancer	6.5	1.9	31.6	$-$9.7	12.2	42.5	6.9
Neoplasms	Lip and oral cavity cancer	6.2	1.8	4.3	3	21.1	36.4	6.8
Neoplasms	Liver cancer	6.6	2.6	23.6	9.8	13.4	56	6.7
Neoplasms	Multiple myeloma	5.5	1.1	5.1	3.2	$-$1.1	13.8	6.4
Neoplasms	Kidney cancer	6.4	2.9	29	5.8	2.1	46.2	6.3
Neoplasms	Uterine cancer	5.4	0.5	$-$1.2	8.8	$-$7.6	5.9	5.2
Neoplasms	Non$-$melanoma skin cancer	5.4	3.8	4.4	$-$9.1	0.2	4.7	4.6
Neoplasms	Malignant skin melanoma	5.9	1.7	4.1	4.8	13	29.5	4.2
Neoplasms	Other pharynx cancer	5.5	2	$-$38.7	4.4	29.3	2.6	3.5
Neoplasms	Cervical cancer	5.8	0.2	$-$4.1	5.9	14.1	21.9	3.2
Neoplasms	Thyroid cancer	6.2	0.5	3.8	7.1	20.8	38.4	3
Neoplasms	Gallbladder and biliary tract cancer	6	3.2	$-$9.1	16.3	13.3	29.7	3
Neoplasms	Mesothelioma	5.6	1.5	$-$4.2	$-$3.6	8.2	7.5	2.8
Neoplasms	Hodgkin lymphoma	5.1	$-$0.5	$-$5.8	0	28.4	27.2	1.5
Neoplasms	Larynx cancer	5.5	2.6	$-$18.3	13.2	12.2	15.2	1.5
Neoplasms	Testicular cancer	6.2	$-$1.2	4.5	9	24.2	42.6	1.2
Neoplasms	Nasopharynx cancer	3	1.3	$-$12.2	1.5	$-$14.1	$-$20.5	0.4
OtherNCD	Endocrine, metabolic, blood, and immune disorders	5.5	0.4	15.5	1.1	$-$12.2	10.29	64.8
OtherNCD	Urinary diseases and male infertility	6.3	2.2	26.8	1.2	6.6	43.1	59.4
OtherNCD	Congenital birth defects	5.5	0.7	$-$6.5	5.8	3.8	9.3	52.5
OtherNCD	Gynecological diseases	5.3	$-$1	$-$6.9	$-$0.4	3.7	0.6	43.8
OtherNCD	Oral disorders	6.5	$-$1.6	36.7	2.9	6.8	51.3	7.9
OtherNCD	Hemoglobinopathies and hemolytic anemias	5.5	1.1	2	3.2	$-$9.2	2.6	4.9
Digestive	Other digestive diseases	5.3	1.3	$-$5.2	$-$0.4	$-$0.5	0.5	61.4
Digestive	Inguinal, femoral, and abdominal hernia	5.6	1.4	6.2	0	0.1	13.2	34
Digestive	Gallbladder and biliary diseases	5.4	1.5	1.5	$-$1.7	$-$1.4	5.4	33.7
Digestive	Appendicitis	5.4	$-$0.4	$-$1.5	$-$1.1	4.6	7	18.8
Digestive	Cirrhosis and other chronic liver diseases	5.2	0.6	$-$12.2	4.7	$-$2	$-$3.7	18.8
Digestive	Paralytic ileus and intestinal obstruction	4.9	2.1	$-$15	1.8	$-$4.9	$-$11.1	17.5
Digestive	Upper digestive system diseases	5.5	1.6	$-$1.7	1	2.3	8.8	14.9
Digestive	Pancreatitis	5.9	0.8	17.9	$-$1	4.3	27.9	9.9
Digestive	Inflammatory bowel disease	5.5	0.2	$-$10.6	5.2	10.7	11.1	7.9
Digestive	Vascular intestinal disorders	6.3	2.2	48	0.6	$-$19.5	37.6	4.1
Chronic respiratory	Other chronic respiratory diseases	5.6	$-$0.1	12.2	$-$0.4	$-$4.9	12.4	35.7
Chronic respiratory	Chronic obstructive pulmonary disease	5.4	2.4	$-$3.8	4.6	$-$1.7	6.9	19.7
Chronic respiratory	Interstitial lung disease and pulmonary sarcoidosis	5.8	2.6	20.9	10.8	$-$13.3	26.7	3.9
Chronic respiratory	Asthma	5.8	1.1	$-$16.1	6.2	24.2	21.2	1.3
Chronic respiratory	Pneumoconiosis	2.3	1.1	$-$9.2	$-$9.2	$-$9.2	$-$24	0.1
Neurological	Epilepsy	5.5	0	3.7	2.1	$-$2.4	8.87	21.5
Neurological	Other neurological disorders	5.9	1.1	$-$1.3	1.4	17.8	24.9	21.4
Neurological	Alzheimer’s disease and other dementias	5.3	3.3	4.1	1.8	$-$13.1	1.3	6.5
Neurological	Parkinson’s disease	5.2	2.7	$-$13.3	3.2	4.44	2.3	5.6
Neurological	Multiple sclerosis	4.5	0.7	$-$12.1	$-$7.2	$-$4.5	$-$18.6	1
Neurological	Motor neuron disease	4.7	2.6	$-$26.2	3.3	$-$1.4	$-$17.1	0.9
Neurological	Headache disorders	6.6	$-$0.4	35.7	4.6	13.6	60	0.8
Skin and subcutaneous	Skin and subcutaneous diseases	5.3	1.4	2.7	$-$0.9	$-$7.1	1.4	36.6
Sense organ	Blindness and vision impairment	5.4	2	$-$14.7	2.8	7.6	3.1	18.2
Sense organ	Other sense organ diseases	5.1	1.3	$-$8.3	$-$1.9	$-$2.8	$-$6.7	9.8
Diabetes	Chronic kidney disease	5	1	$-$18.3	1.4	2.8	$-$8.2	12.8
Diabetes	Diabetes mellitus	6.4	2.3	17.3	6.3	16.6	48.9	11.4
Diabetes	Acute glomerulonephritis	4.9	2.2	58.3	58.3	58.3	182	0.1
Substance use	Alcohol use disorders	5.4	0.4	4.8	4.9	$-$9.3	6.2	6
substance use	Drug use disorders	4.8	$-$1.1	22.8	$-$2.1	$-$44	$-$19.6	3
Mental	Mental disorders	5.8	2.1	0.6	2.2	11.6	22.3	27.6
Communicable	Maternal and neonatal disorders	5.8	0.3	13	1.7	$-$1.2	19.5	104.6
Communicable	Other infectious diseases	5.5	2	$-$32	$-$0.6	31.6	6.4	89.8
Communicable	Respiratory infections and tuberculosis	6	2.7	24.9	0.2	$-$6	27.8	52.1
Communicable	Enteric infections	5.5	2	0.5	$-$0.9	4.6	11.7	11.7
Communicable	Nutritional deficiencies	5.3	2.2	$-$6.8	$-$1.4	4.9	4.3	3.8
Communicable	HIV/AIDS and sexually transmitted infections	4.9	$-$0.9	$-$10.9	0.4	0.9	$-$5.5	2.9
Communicable	Neglected tropical diseases and malaria	5.9	$-$1.3	$-$11.4	6.1	27	26.4	0.9
Injuries	Injury of one body part	5.6	1.5	$-$3.6	0.9	9	13.4	257.7
Injuries	Injury of multiple body parts	6.3	1.5	28.8	1.5	3.7	41.8	88.4
Injuries	Unintentional injuries	4.8	0.9	$-$15.2	0.7	$-$9.7	$-$18.6	12.5
Well care	Well care	5.4	$-$0.1	$-$3.2	$-$0.4	3.3	5	115.7
Non-distinctive	Non-distinctive codes	5	2	$-$9.8	0.2	$-$8.5	$-$11.2	46.1

## Footnotes

1 The six non-listed hospitals that do not get governmental co-payments are not included since their financing is completely outside of the social health insurance system and the case costs are not provided to the cantonal authorities. The number (share) of total cases in these hospitals was 15,196 (6.8%) in 2013 and 14,165 (5.9%) in 2017. According to key data on the Swiss hospitals, the aggregate costs (share) amounted to 201.0 m CHF (7.0%) in 2013 and 207.5 m CHF (6.4%) in 2017 [27]. Hence the costs analyzed in this paper represent 93% (2013) and 93.6% (2017) of total inpatient costs.

2 We proceeded in the same way as [16] and [14] in their decomposition of health care spending by diseases for Switzerland and the US. Besides the classification of disease groups, these studies defined additional conditions such as "well care" and the remaining category of non-distinctive codes to make the classification fully comprehensive. The use of the same disease classification as previous studies guarantees comparability.

3 Mental disorders are not usually treated in the acute inpatient setting considered here and skin and subcutaneous diseases showed low number of observations at the GBD level 3.

4 The specification is then:

$$\begin{aligned} costs&=\sum _{d=1}^{100} \sum _{a=1}^{42}population*\frac{population_{a}}{population}* \frac{treated\,patients_{a,d}}{population_{a}}\\ &\quad* \frac{stays_{a,d}}{treated\,patients_{a,d}}*\frac{costs_{a,d}}{stays_{a,d}} \end{aligned}$$

5 For few diseases with low costs such as HIV/AIDS and sexually transmitted infections, the specification that was chosen for the distributional analysis yielded a different sign for the factor’s association with costs. This is due to the fact that in the second specification, the overall effect is the sum over five distribution sections, whereas in the first specification, it is the sum over 42 (smaller) age/sex groups. Absolute differences are very small, however.

6 Moreover, the estimates are only partially reasonable, which is mainly because they are not in every case based on Swiss data, but inferred from data from neighbouring countries. One example is the prevalence rate of diabetes in 2017, which is estimated at 11.1%. This is too high compared to recent evidence [40, 41]. Second, they are not available at the sub-national level.

7 Only a part of costs (19.3% in 2017) represented direct costs, i.e. cost items directly assigned to the case (e.g. physicians’ time with the patient and drugs). The rest was fixed costs from services that are shared by all patients (e.g. operating rooms and imaging). However, it is important to note that the term “fixed” does not mean that costs are simply apportioned across cases. The share allocated to each case is closely linked to its actual resource use. Fixed costs are assigned based on the time the service was used (e.g. operation room) or other measures of intensity. Hospitals also have the possibility to weight the costs by disease severity or service use intensity prior to allocation. Hospital accounting experts confirmed that the cost allocation clearly reflects actual resource use for most cost types, which is also in line with the primary goal of the accounting, namely the reporting of case-specific costs for DRG calculations.

## References

[1] Federal Statistical Office: Kosten und Finanzierung des Gesundheitswesens (National Health Accounts) (2018)

[2] Federal Statistical Office: Krankenhausstatistik (Hospital Statistics) (2018)

[3] World Bank: World Bank Open Data. https://data.worldbank.org/ (2019). Accessed 1 Oct 2020

[4] Das Gupta, P.: Standardization and decomposition of rates: a user’s manual. US Department of Commerce, Economics and Statistics Administration, Bureau of the Census (1993)

[5] Dieleman JL, Squires E, Bui AL, Campbell M, Chapin A, Hamavid H, Horst C, Li Z, Matyasz T, Reynolds A (2017). Factors associated with increases in US health care spending, 1996–2013. J. Am. Med. Assoc..

[6] De Pietro C, Camenzind P, Sturny I, Crivelli L, Edwards-Garavoglia S, Spranger A, Wittenbecher F, Quentin W (2015). Switzerland: health system review. Health Syst. Trans..

[7] Getzen TE (1992). Population aging and the growth of health expenditures. J. Gerontol..

[8] Zweifel P, Felder S, Meiers M (1999). Ageing of population and health care expenditure: a red herring?. Health Econ..

[9] Bodenheimer T (2005). High and rising health care costs. Part 2: technologic innovation. Ann Internal Med..

[10] Smith S, Newhouse JP, Freeland MS (2009). Income, insurance, and technology: why does health spending outpace economic growth?. Health Aff..

[11] Colombier C (2018). Population ageing in healthcare-a minor issue? Evidence from Switzerland. Appl. Econ..

[12] Hartwig J (2008). What drives health care expenditure?—Baumol’s model of ‘unbalanced growth’ revisited. J. Health Econ..

[13] Colombier C (2017). Drivers of health-care expenditure: what role does Baumol’s cost disease play?. Soc Sci Q..

[14] Dieleman JL, Baral R, Birger M, Bui AL, Bulchis A, Chapin A, Hamavid H, Horst C, Johnson EK, Joseph J (2016). US spending on personal health care and public health, 1996–2013. J. Am. Med. Assoc..

[15] Kinge JM, Sælensminde K, Dieleman J, Vollset SE, Norheim OF (2017). Economic losses and burden of disease by medical conditions in Norway. Health Policy..

[16] Wieser S, Riguzzi M, Pletscher M, Huber CA, Telser H, Schwenkglenks M (2018). How much does the treatment of each major disease cost? A decomposition of Swiss National Health Accounts. Eur. J. Health Econ..

[17] Dunn, A., Liebman, E.B. and Shapiro, A.: Decomposing medical-care expenditure growth. NBER Working Paper No. 23117 (2017)

[18] Dunn A, Rittmueller L, Whitmire B (2016). Health care spending slowdown from 2000 to 2010 was driven by lower growth in cost per case, according to a new data source. Health Aff..

[19] Thorpe KE (2013). Treated disease prevalence and spending per treated case drove most of the growth in health care spending in 1987–2009. Health Aff..

[20] Roehrig CS, Rousseau DM (2011). The growth in cost per case explains far more of US health spending increases than rising disease prevalence. Health Aff..

[21] Starr M, Dominiak L, Aizcorbe A (2014). Decomposing growth in spending finds annual cost of treatment contributed most to spending growth, 1980–2006. Health Aff..

[22] Cutler DM, Ghosh K, Messer KL, Raghunathan TE, Stewart ST, Rosen AB (2019). Explaining the slowdown in medical spending growth among the elderly, 1999–2012. Health Aff..

[23] Zhai T, Goss J, Li J (2017). Main drivers of health expenditure growth in China: a decomposition analysis. BMC Health Serv. Res..

[24] Wong A, van Baal PHM, Boshuizen HC, Polder JJ (2011). Exploring the influence of proximity to death on disease-specific hospital expenditures: a carpaccio of red herrings. Health Econ..

[25] Pellegrini, S., Roth, S.: Évolution des coûts et du financement dans le système de soins depuis la révision du financement hospitalier. Obsan Rapport **73**, (2018)

[26] Tuch, A., Joerg, R., Hedinger, D., Widmer, M.: Qualität der stationären Leistungen unter der neuen Spitalfinanzierung. Monitoring der Qualitätsindikatoren 2009–2016. Obsan Dossier 65 (2018)

[27] Federal Office of Public Health: Kennzahlen der Schweizer Spitäler (Key data on Swiss hospitals) (2015–2019)

[28] Statistical Office: Daten Bevölkerungsbestand. https://statistik.zh.ch/internet/justiz\_inn eres/statistik/de/daten/daten\_bev oelkerung\_soziales/bevoelkerung.html (2019). Accessed 1 Oct 2020

[29] H+: REKOLE^®^. https://www.hplus.ch/de/rechnungswesen/handbuch-rekole (2019). Accessed 1 Oct 2020

[30] GBD (2018). 2017 Causes of Death Collaborators: Global, regional, and national age-sex-specific mortality for 282 causes of death in 195 countries and territories, 1980–2017: a systematic analysis for the Global Burden of Disease Study 2017. The Lancet..

[31] Rosen AB, Aizcorbe A, Highfill T, Chernew ME, Liebman E, Ghosh K, Cutler DM, Aizcorbe A, Baker C, Berndt ER, Cutler DM (2016). Attribution of health care costs to diseases: does the method matter?. Measuring and modeling health care costs.

[32] Chevan A, Sutherland M (2009). Revisiting Das Gupta: refinement and extension of standardization and decomposition. Demography..

[33] Oaxaca, R.: Male-female wage differentials in urban labor markets. Int. Econ. Rev. 693–709 (1973)

[34] Blinder, A.S.: Wage discrimination: reduced form and structural estimates. J. Human Resour. 436–455, (1973)

[35] Li J (2017). Rate decomposition for aggregate data using Das Gupta’s method. Stata J..

[36] Roth, M., Roth, S.: Entwicklung der Ausgaben der obligatorischen Krankenpflegeversicherung von 1998 bis 2010. Obsan Dossier **53**, (2012)

[37] Krämer J, Schreyögg J (2019). Demand-side determinants of rising hospital admissions in Germany: the role of ageing. Eur. J. Health Econ..

[38] Cutler DM, McClellan M (2001). Is technological change in medicine worth it?. Health Aff..

[39] Department of Health: Veröffentlichungen - Kenndaten Akutsomatik. https://gd.zh.ch/internet/gesundheitsdirektion/de/unsere\_direktion/veroeff entlichungen.html (2019). Accessed 1 Oct 2020

[40] Kaiser A, Vollenweider P, Waeber G, Marques-Vidal P (2012). Prevalence, awareness and treatment of type 2 diabetes mellitus in Switzerland: the CoLaus study. Diabet. Med..

[41] Bopp M, Zellweger U, Faeh D (2011). Routine data sources challenge international diabetes Federation extrapolations of national diabetes prevalence in Switzerland. Diabet. Care..

[42] Roth, S., Pellegrini, S.: Virage ambulatoire: transfert ou expansion de l’offre de soins? Obsan Rapport **68**, (2015)

[43] Dafny LS (2005). How do hospitals respond to price changes?. Am. Econ. Rev..

[44] Kutz A, Gut L, Ebrahimi F, Wagner U, Schuetz P, Mueller B (2019). Association of the Swiss diagnosis-related group reimbursement system with length of stay, mortality, and readmission rates in hospitalized adult patients. JAMA Netw Open..

[45] Busato A, von Below G (2010). The implementation of DRG-based hospital reimbursement in Switzerland: a population-based perspective. Health Res. Pol. Syst..

[46] Maeda JLK, Raetzman SO, Friedman BS (2012). What hospital inpatient services contributed the most to the 2001–2006 growth in the cost per case?. Health Serv. Res..

[47] Jones AM, Lomas J, Rice N (2015). Healthcare cost regressions: going beyond the mean to estimate the full distribution. Health Econ..

[48] De Meijer C, O’Donnell O, Koopmanschap M, Van Doorslaer E (2013). Health expenditure growth: looking beyond the average through decomposition of the full distribution. J. Health Econ..

[49] Rizzo JA, Chen J, Gunnarsson CL, Naim A, Lofland JH (2015). Adjusting for comorbidities in cost of illness studies. J. Med. Econ..

[50] Dieleman JL, Baral R, Johnson E, Bulchis A, Birger M, Bui AL, Campbell M, Chapin A, Gabert R, Hamavid H (2017). Adjusting health spending for the presence of comorbidities: an application to United States national inpatient data. Health Econ. Rev..

